# Evaluation of the Safe Care, Saving Lives (SCSL) quality improvement collaborative for neonatal health in Telangana and Andhra Pradesh, India: a study protocol

**DOI:** 10.1080/16549716.2019.1581466

**Published:** 2019-03-08

**Authors:** Claudia Hanson, Karen Zamboni, Vikrant Prabhakar, Ajitkumar Sudke, Rajan Shukla, Mukta Tyagi, Samiksha Singh, Joanna Schellenberg

**Affiliations:** a Department of Disease Control, London School of Hygiene and Tropical Medicine, London, UK; b Department of Public Health Sciences, Karolinska Institutet, Stockholm, Sweden; c Department of Community Medicine, Adesh Medical College and Hospital, Kurukshetra, India; d ACCESS Health International, Hyderabad, India; e Public Health Foundation of India, Kavuri Hills, Madhapur, Hyderabad, India

**Keywords:** Collaborative quality improvement, perinatal health, India, evidence-based practices, neonatal mortality, stillbirth, implementation science

## Abstract

**Background**: The collaborative quality improvement approach proposed by the Institute for Healthcare Improvement has the potential to improve coverage of evidence-based maternal and newborn health practices. The Safe Care, Saving Lives initiative supported the implementation of 20 evidence-based maternal and newborn care practices, targeting labour wards and neonatal care units in 85 public and private hospitals in Telangana and Andhra Pradesh, India.

**Objective**: We present a protocol for the evaluation of this programme which aims to (a) estimate the effect of the initiative on evidence-based care practices and mortality; (b) evaluate the mechanisms leading to changes in adherence to evidence-based practices, and their relationship with contextual factors; (c) explore the feasibility of scaling-up the approach.

**Methods**: The mixed-method evaluation is based on a plausibility design nested within a phased implementation. The 29 non-randomly selected hospitals comprising wave II of the programme were compared to the 31 remaining hospitals where the quality improvement approach started later. We assessed mortality and adherence to evidence-based practices at baseline and endline using abstraction of registers, checklists, observations and interviews in intervention and comparison hospitals. We also explored the mechanisms and drivers of change in adherence to evidence-based practices. Qualitative methods investigated the mechanisms of change in purposefully selected case study hospitals. A readiness assessment complemented the analysis of what works and why. We used a difference-in-difference approach to estimate the effects of the intervention on mortality and coverage. Thematic analysis was used for the qualitative data.

**Discussion**: This is the first quality improvement collaborative targeting neonatal health in secondary and tertiary hospitals in a middle-income country linked to a government health insurance scheme. Our process evaluation is theory driven and will refine hypotheses about how this quality improvement approach contributes to institutionalization of evidence-based practices.

## Background

Every year around 760,000 babies die in India in the first 28 days of life, and in 2018 the neonatal mortality was estimated to be high at 24 deaths per 1000 live births [1] with wide variations within the country. To address neonatal mortality, the Indian government recently made important investments to make care during childbirth and in the first few days of life more accessible. The Janani Suraksha Yojana (JSY) cash transfer scheme was set up in 2005 to encourage women to deliver in health facilities [2], and the Janani Shishu Suraksha Karyakram (JSSK) scheme provides free treatment, food and transport for women to access childbirth care or to reach care for sick newborns [3].

Since 2014, three levels of neonatal care have been established: (1) Newborn Care Corners at all points of childbirth, providing essential care at birth, including resuscitation; (2) Level-I Newborn Stabilization Units providing management of low birthweight babies not requiring intensive care, and stabilization of sick newborns before further referral; (3) Level-II care in Special Newborn Care Units at district and sub-district hospitals providing all types of care to sick newborns, except assisted ventilation and surgeries; and (4) Level-III Neonatal Intensive Care Units [4,5].

The recent investments in health infrastructure and access to care heighten the importance of improving quality of care and adherence to best practices. Several studies from India have indicated deficits in quality of care beyond infrastructural deficits [6–8]. Although training is an important intervention to provide good quality of care, in-service training is likely to yield limited returns if done in isolation; hence quality management and quality improvement approaches are increasingly required to support high-quality services.

To reduce neonatal mortality and stillbirths and improve quality of newborn care, in 2014 ACCESS Health International (ACCESS) with support from the Institute of Healthcare Improvement started the Safe Care, Saving Lives (SCSL) initiative in two states of India, Telangana and Andhra Pradesh, based on the Breakthrough Series Quality Improvement Collaborative (QIC) approach developed by the Institute of Healthcare Improvement [9].

The QIC approach aims to improve adherence to evidence-based practices (EBP) in health care settings, through the use of structured quality improvement methods and collaboration with other participating teams working on similar issues [9,10]. Health facility teams are supported to take a problem-solving approach, through training or sensitization in quality improvement methodologies and coaching and mentoring by external staff, and are encouraged to learn from other active teams during so-called learning sessions. This approach is increasingly employed in low-resource settings to improve quality of care [11], scale-up interventions [12–14] or improve quality of care with a health system strengthening objective [15–17]. The recently launched quality of care networks in Africa and India are also based on this approach [18,19]. The approach is typically implemented in phases, called waves, where the first wave often constitutes a piloting period to refine the approach and summarize innovations and changes to improve compliance with EBP.

The Safe Care, Saving Lives programme targeted secondary and tertiary newborn care units and the labour ward. These were public Special Newborn Care Units (level-II facilities), and both public and private Neonatal Intensive Care Units (level-III facilities), in hospitals empanelled into two state insurance schemes covering care for severely sick babies, in Telangana and Andhra Pradesh. In this paper we use the term ‘newborn care unit’ to include both Special Newborn Care Units and Neonatal Intensive Care Units.

Despite the increasing application of the QIC approach, evidence on its effectiveness is limited and inconclusive [20]. Very few robust studies evaluating QIC are available from low- or middle-income countries [14,17]. In addition, the Safe Care, Saving Lives initiative used an innovative approach of nesting the QIC within a Health Care Trust platform which could potentially act as an external driver of quality improvement. Responding to the importance of providing more evidence on the effectiveness of the QIC approach and how it affects outcomes, we aim to assess:

1. The effect of the Safe Care, Saving Lives initiative on essential maternal and newborn evidence-based care practices, the stillbirth rate and the neonatal mortality in labour wards and neonatal care units
2. The mechanisms leading to changes in adherence to evidence-based care practices, and their relationship with contextual factors
3. The feasibility of scaling-up the QIC approach through a government-sponsored health insurance platform.

## Methods

### Study design

The evaluation is based on a plausibility design nested within a phased implementation approach where the 29 non-randomly selected hospitals comprising wave II (March 2017 – August 2018) of the programme are compared to the 31 hospitals where the QIC approach will start as a part of wave III in consultation with the Government (Table 1). We use a mixed methods design, employing quantitative and qualitative approaches to assess mortality and adherence to EBP, and to explore the mechanisms and drivers of change in adherence to EBP.

**Table 1.** Timeline.

Safe Care, Saving Lives Programme timeline												
	Year											
					2017				2018			
Phase	2013	2014	2015	2016	Jan–Mar	Apr–Jun	Jul–Aug	Sep–Dec	Jan–Mar	Apr–Jun	Jul–Aug	Sep–Dec
Programme design phase												
Design of Quality Improvement toolkit												
Wave 1 (25 hospitals)												
Programme review and adaptation of design												
Wave 2 (29 hospitals)												
Wave 3 (31 hospitals)*												
Baseline data collection				X								
Endline data collection											X	

*wave 3 is currently on hold.

### Study sites

Telangana and Andhra Pradesh – a new state which was formed in 2014 when Andhra Pradesh split into two – are situated in Southern India and are characterized by slightly better socio-economic development indicators than the Indian average (Web annex A) [21]. The neonatal mortality rate was estimated in 2015 at 12 in urban and 33 in rural areas [22]. As in other parts of India, prematurity, low birthweight and intrapartum-related complication/asphyxia together with neonatal infection are the main reasons why babies die [23].

**Figure 1.**
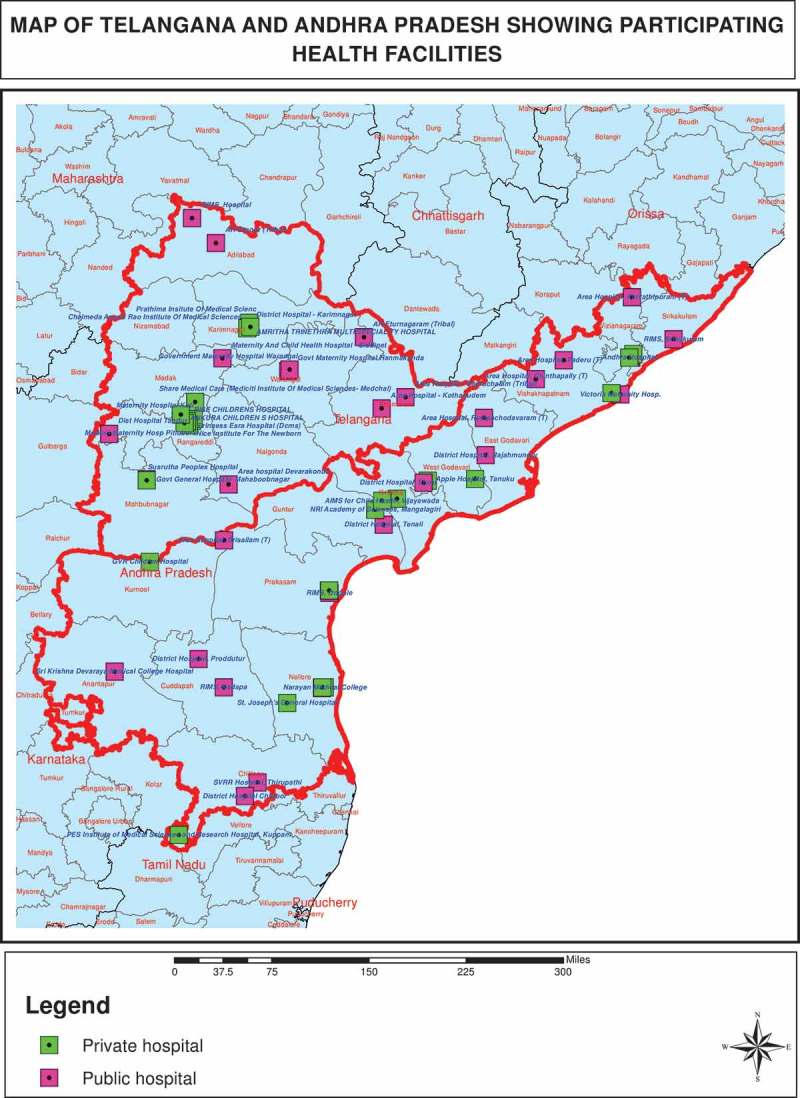
The study area.

The study population consists of all 85 private and public hospitals with a newborn care unit (level II or III) that are empanelled with the government-sponsored health insurance schemes: the Aarogyasri Health Care Trust in Telangana and the Dr Nandamuri Taraka Rama Rao Vaidya Seva in Andhra Pradesh (Figure 1). The schemes provide poor families with access to secondary and tertiary care in private and public hospitals [24], covering care for surgical and medical conditions, including cancer care, cardiac treatment, neurological diseases and trauma care. In the area of maternal and newborn care, the health care trusts cover expenses for septicaemia in need of third line antibiotic treatment, stabilization and care for babies with malformations and ventilation [25–27]. Approximately 70% of the population is eligible for the health insurance cover. In order to qualify for service reimbursement from the Health Care Trust, hospitals have to fulfil defined infrastructure and treatment conditions [28].

Of the 85 target hospitals, 25 (10 private and 15 public) across Andhra Pradesh (10) and Telangana (15) were enrolled in the programme wave I, where the approach was piloted and refined. These 25 were excluded from this study. The 60 hospitals of wave II and wave III comprised 35 hospitals in Andhra Pradesh and 25 in Telangana, of which 26 and 34 were private and public facilities, respectively. Of the 26 private facilities, only 15 had a labour room. The public facilities included teaching hospitals with a large patient load under the control of the Directorate of Medical Education, and non-teaching hospitals (district and sub-district) which are under control of the Commissioner, Vaidya Vidhana Parishad in both states.

At baseline (2016), all except three public facilities operating at district or sub-district level were equipped with a newborn care unit (Special Neonatal Care Unit) and provided level II care for the sick newborns. All private hospitals and medical colleges were equipped with Neonatal Intensive Care Units (Level II and III). Three facilities were in the process of upgrading their services from Newborn Stabilization Units to level II neonatal care facilities.

The caseload in private facilities as assessed during our baseline survey was generally much lower than in public facilities. The median number of deliveries in private facilities was 39 (mean 99) in a 1-month period, while a median of 315 babies (mean 328) were born per month in public facilities (Table 2).

**Table 2.** General information on hospitals included.

	Explanation of those missing	Telangana N = 25	Andhra Pradesh N = 35	Public N = 34	Private N = 26
Refused participation in baseline assessment		4 private	3 public & 1 private college	3 public facilities	5 private
Have a neonatal care unit		**21**	**31**	**31**	**21**
Facility assessment done in neonatal care unit	3 missing ~	21*	**28**	**30**	**19**
Mean/median (IQR) no. of admission per month		127/106 (47–152)	90/58 (31–121)	130/106 (67–170)	67/41 (22–53)
Have a breastfeeding room	1 missing	15 (71%)	26 (93%)	25 (83%)	16 (84%)
Have a Kangaroo Mother Care (KMC) room	5 missing	13 (62%)	13 (46%)	17 (57%)	9 (47%)
Mean/median		1.9/1	2.3/1	1.4/1	3.3/2
(IQR) no. of paediatricians working during the day-shift		(1−2)	(1–3)	(1–1)	(1–4)
Have a labour room	12 hospitals without labour ward, one refused data collection	**15**	**24**	**30**	**9**
Facility assessment done in labour room		**15**	**24**	**30**	**9**
Mean/median	3 missing information on mean no. of deliveries	348/267	229/197	328/315	99/39
(IQR) of no. of deliveries per month		(99–382)	(81–338)	(166–400)	(29–177)
Working 24 × 7 operation theatre for Caesarean section		13 (87%)	21 (88%)	25 (83%)	9 (100%)
Mean (IQR) no. of obstetricians working during the day-shift		.9/2	3.0/2	2.8/2	3.3/2
		(1–5)	(1–3.5)	(1−4)	(2–5)

Source: Facility checklists ~ 1 SNCU/NICU register were not available due to reconstruction of the SNCU/NICU; and two SNCU/NICU data could not be retrieved as the data base was not maintained. *Three of the 21 neonatal care units were Newborn Stabilization Units (NBSU) in process of upgrading at the time of assessment.

Similarly, while the median number of babies admitted to a newborn care unit over a 1-month period in private facilities was 41 (mean 67), more than twice as many were admitted in public facilities (median 106, mean of 130). Only 83% (25 of 30) of public hospitals providing delivery care had a functional operating theatre to perform Caesarean sections. The number of obstetricians and paediatricians was slightly higher in private than public facilities: median 2 (mean 3.3) in private and median 2 (mean 2.8) obstetricians in public facilities, and median 2 (mean 3.3) in private and median 1 (mean 1.4) paediatrician in public facilities. The effect of this difference on care was exacerbated by the fact that the caseload was much lower in private than public facilities.

### The Safe Care, Saving Lives intervention

The project was based on the QIC approach developed by the Institute for Healthcare Improvement [9], and the Model for Improvement (Web annex B) described by Langley and colleagues [29]. The approach has the following features:

- A focused clinical subject
- Learning from experts in fields of obstetrics, neonatology and quality improvement
- Using the Model for Improvement and multiple Plan-Do-Study-Act (PDSA) cycles
- Quality Improvement Teams collect data to measure their performance
- Collaborative learning sessions between hospitals.

The EBP that the Safe Care, Saving Lives programme focused on were first identified and prioritized in 2014 by an expert team comprising neonatologists, obstetricians, quality improvement specialists and a member of the World Health Organization, and then updated in March 2016. Overall, they identified 20 EBP which were not consistently implemented in Indian hospitals and which, if implemented at scale, would be expected to improve neonatal morbidity and mortality. These EBP addressed the three main drivers of neonatal mortality in India as well as globally and relate to: (1) reliable intra-partum care and newborn resuscitation, (2) prevention and management of complications from prematurity, and (3) neonatal sepsis prevention and management [30]. The topics include interventions in the labour room and the neonatal care unit (Table 3). Most of the 20 focus EBP were summarized in a Quality Improvement Toolkit, which described the practices, measurement indicators and audit tools, and suggested 73 possible change strategies to test, based on successful experience elsewhere.

**Table 3.** Evidence-based practices included in the Safe Care, Saving Lives initiative.

	Sepsis bundle	Prematurity bundle	Asphyxia bundle
Practices promoted in labour rooms	Antibiotics to women at risk of sepsis Hand hygiene & gloves during per-vaginal examination WHO 6 cleans	Ante-natal steroids Early breastfeeding	High risk categorization of woman in labour Trained personnel for high risk delivery Compliance with partogram Pre-delivery checklist Compliance with oxytocin infusion protocol Resuscitation with bag and mask
Practices promoted in neonatal care units	Hand hygiene Protocol for central vascular catheter Aseptic Peripheral IV line insertion Antibiotics to neonates born to mother with risk factors for sepsis Prevent ventilator associated Pneumonia	First temperature in 15 minutes from admission Exclusive breastfeeding Kangaroo Mother Care	CPAP in preterm neonates with respiratory distress
Total no. of practices	**8**	**5**	**7**

The intervention brought together Quality Improvement Teams from several facilities to work on common problems. Each Quality Improvement Team was trained and coached to use the Model for Improvement, a structured approach to identify aims of the improvement process, measures of success and key causes of suboptimal performance; to identify specific changes to improve care, drawing from the Quality Improvement Toolkit or developing new ideas; and to test changes through PDSA cycles and using data to review what worked and what did not.

Quality Improvement Teams also participated in so-called collaborative learning sessions bringing together teams from different hospitals working on a similar issue, to share experiences and lessons learned from quality improvement efforts (Web annex B).

### Implementation strategy

The programme was implemented by ACCESS [31] an international non-governmental organization with some technical support from the Institute of Healthcare Improvement [32]. The programme intervened at three interconnected levels (Web annex B):

- At the level of individual participating hospitals, where hospitals implemented quality improvement activities in newborn care units and (if available) labour rooms.
- At the collaborative level, where groups of hospitals shared learning and experience of quality improvement including clinical knowledge.
- At the state health system level, where the programme engaged institutional stakeholders to promote and prioritize quality improvement.

Each hospital was supported by a Quality Improvement Mentor and a Senior Associate or Quality Improvement Lead. Using the Model for Improvement, Associates/Leads also worked with the hospital leadership to promote their engagement in quality improvement and help facilitate the removal of bottlenecks to the adoption of EBP (Web annex B).

The intervention at the level of hospitals aimed to improve institutionalization of EBP by strengthening capacity and leadership for continuous quality improvement, and by supporting changes in team work, accountability and organization of care which, together, were assumed to improve cooperation between relevant departments and cadres, and generate social norms supporting the adherence to new EBP.

Collaborative activities across hospitals were assumed to accelerate the pace of improvement, by tailoring innovation to the context; demonstrating the feasibility of implementing quality improvement in similar settings; and activating normative pressures on participating hospitals. The Breakthrough Collaborative approach, entailing three to four gatherings of all participating facilities, proved inefficient during wave I. In wave II, it involved virtual or face-to-face mini-collaborative *learning sessions* among wave I and II facilities working on the same evidence-based practice, mostly consisting of a referral hospital with its referring facilities, and a good performing wave I hospital participating as a model.

The programme envisaged the government-sponsored health care trusts taking over the facilitation and coordination of collaborative learning sessions and dissemination of success stories at the end of the intervention in each State.

At the level of the State health system, the Safe Care, Saving Lives programme provided technical assistance for the creation of a Quality Improvement Unit within the Aarogyasri Health Care Trust to provide an ongoing quality control function for empanelled hospitals, and for the development of an incentive system to link insurance payments to quality improvement measures.

Furthermore, the programme provided capacity building and technical support to the State Quality Assurance Committee and District Quality Assurance Managers, to improve operationalization of quality improvement methods recommended in the National Quality Assurance System [33], and greater prioritization of quality improvement during quality assurance monitoring visits. This component of the programme was added at the end of wave I, recognizing that upstream interventions were necessary to promote engagement of hospital leadership on quality improvement.

### Evaluation strategy

The evaluation was led by a research team comprising researchers from the Public Health Foundation of India (PHFI) and the London School of Hygiene and Tropical Medicine (LSHTM). For the quantitative evaluation we compared impact and outcome measures reflecting the improvement topics of 29 intervention hospitals (wave II) with 31 comparison hospitals (wave III). The data were collected during a baseline (June to August 2016) and endline assessment (August to October 2018) (Table 1). At baseline, only 23 wave II and 29 wave III facilities participated in the survey and only these were considered for the endline. We defined three impact indicators: (1) the stillbirth rate (number of foetuses born without any signs of life and weighing 1000 g or more, of all births) which should reflect the evidence-based better practices for reliable intra-partum care and newborn resuscitation; (2) 7-day; and (3) 28-day neonatal mortality after admission to neonatal care unit (babies who died before completed 7/28 days of life per all babies admitted to neonatal care unit) which should primarily reflect the effect of preventing complications from prematurity and neonatal sepsis. We defined several output indicators reflecting the EBP (Web annex C).

Our quantitative baseline survey used 18 data collection tools including (i) labour room and newborn care unit checklists to investigate infrastructure, supplies and human resources; (ii) abstraction of case notes and observations to investigate implementation of the 20 EBP; and (iii) abstraction of registers in labour wards and newborn care units complemented by on-site interviews with mothers and telephonic follow-up of mothers to estimate mortality after discharge for babies from labour rooms and newborn care units.

The facility checklists were drafted based on standards for labour room and newborn care services in India [34,35]. The observation tools were informed by internationally recommended checklists [36–38] and international standards in hygiene and handwashing. We designed tools to abstract case notes and register information in response to the defined indicators and reflecting international clinical guidelines [39]. The interview guidelines with mothers used an established sequence of questions to investigate breastfeeding in newborn care practices [40]. All tools were programmed using native and SQLite application and linked with a backend server database. Android based tablets (Lenovo) were used for data collection and upload. The application used skips and ranges to improve quality of data.

Data collectors with a nursing or public health background were trained for four days before the first pilot and in two rounds of training of three days each before data collection. Data collection was undertaken by six teams, each composed of one supervisor, one field lead/data abstractor, two observers (one labour room and one neonatal care units) and one interviewer. Four observers trained in interviewing skills were responsible for telephonic interviews. The team visited each hospital for a period of six days to collect data. Collected data were saved daily and uploaded on a safe server weekly. The data was extracted in Excel and checked on a weekly basis. The endline assessment used the same approach.

The implementation strength was monitored by the ACCESS team and shared quarterly with the evaluation team, using indicators on the number of EBP promoted in the labour wards and neonatal care units, the number of change ideas tested, the number and frequency of mentoring contacts, training sessions provided and learning sessions held.

The evaluation team documented contextual factors, such as changes in infrastructure, supplies, trainings provided or current programmes to improve quality of maternal and newborn care in target hospitals, through monthly calls with ACCESS staff and mentors.

A mixed methods process evaluation explored how, for whom and under what circumstances the intervention improved compliance with EBP using the Medical Research Council Process evaluation framework [41]. A before and after study in 23 consenting wave II facilities tested the hypothesis that compliance with EBP would improve more in hospitals that had higher readiness for quality improvement at baseline. A qualitative study using a theory-driven multiple case study design explored adaptations of the approach to the context, and participants’ engagement with the quality improvement intervention, using semi-structured interviews of Quality Improvement Mentors, Quality Improvement Teams, hospital leaders, and health workers not involved in quality improvement activities, and non-participant observation of programme activities. A programme theory of change was developed using participatory qualitative evaluation methods (Web annex B) [42,43] and was used as a framework for the qualitative analysis, using data collected in March 2018 and at endline to test hypothesized mechanisms of change and their relationship to the context. Finally, a qualitative study explored the feasibility of scaling-up the QIC approach using government-sponsored health insurance trusts, through semi-structured interviews with programme designers and implementers, and representatives of health insurance trusts, district and state health authorities, and other development partners active in quality improvement.

### Sample size

Our sample size was based on the 3 primary impact indicators of the stillbirth rate in the labour ward and 7-day/28-day neonatal mortality after admissions to the newborn care unit and 10 output indicators. We used the formula proposed by Hayes and Moulton for unmatched clusters [44].

We used estimates from our baseline assessment for the stillbirth rate and neonatal mortality rate and estimates of the implementation of the EBP in the comparison group. We estimated the k factor using our baseline results for four indicators of neonatal mortality, stillbirth rate, high risk admission (based on abstraction of case notes) and handwashing (based on observations).

The number of clusters per arm was fixed at 20 based on the refusal rates experienced during the baseline and due to the fact that 11 of the 60 hospitals did not have a labour ward. We fixed the type 1 error assuming a 5% significance level. We reviewed the potential improvements in mortality and implementation of EBP we may achieve. Table 4 indicates a realistic scenario for each of the indicator having the cluster size fixed at 20 clusters in each arm, and an achievable cluster size per hospital. We aimed to detect a seven-day mortality reduction of 20%. Reaching a cluster size of 95 phone interviews per cluster, we would have only 50% power to be able to indicate a 20% reduction in mortality. However, if we increased our sample to 190 by using register and phone interview data, we would increase our power to 80%. We aimed to include at least 260 observations from registers (births in the last month) in each facility, which would allow us to detect a 35% reduction in stillbirth rate. If we reached a sample of 420 observations (deliveries in the last 1.5 months) we would have 70% power to estimate a smaller reduction of 25%.

**Table 4.** Sample size calculation for the impact and output indicators.

Indicator	Power to detect expected change	Baseline estimates	k-factor	Cluster size	Expected change (%)
7-day mortality (register and phone interviews)	50%	9.0%	0.0001	95	20%
	80%			190	
28-day mortality (register and phone interviews)	52%	9.5%	0.0001	95	20%
Stillbirths (registers)	70%	2.1%	0.01	420	25%
	80%			260	35%
High risk admission (case sheets)	80%	61%	0.01	20	16%
Partograph use (case sheets)	80%	10%	0.01	20	70%
Pre-delivery checklist (case sheets)	80%	30%	0.01	20	32%
Exclusive breastfeeding (phone interview)	80%	93%	0.01	95	3%
Temperature measurement (observations)	80%	52%	0.24	12	35%
Hygienic vaginal examination (observation)	80%	25%	0.24	12	57%
Six cleans in labour ward (observations)	80%	6%	0.24	8	170%
Hygienic cannulations/iv line insertion (observations)	80%	5%	0.24	8	195%
Handwashing	80%	22%	0.24	19	50%
Kangaroo Mother Care	80%	5%	0.24	7	210%

*a priori estimate as Kangaroo Mother Care was not introduced at baseline

For each of the output indicators, we calculated realistic scenarios, setting the power at 80% and the significance at a 95% level. We aimed to abstract 20 case notes in each facility, allowing us to indicate a 16% improvement in detecting and flagging high risk admission (from 61% to 71%), a 70% improvement in partograph use (from 10% to 17%) and a 32% improvement in using a delivery checklist (from 30% to 40%). With 95 telephonic interviews with mothers after admission to the neonatal ward, we would be able to calculate a 3% improvement in exclusive breastfeeding (from 93% to 95%).

### Allocation to intervention and comparison group

The allocation of 60 eligible facilities to intervention and comparison group was done in August 2016. We first stratified the hospitals with regard to the case-fatality category in neonatal care units, their location and their caseload. The strata are explained below. Within the strata we randomly allocated the hospitals to intervention and comparison groups, but this allocation was then adjusted to allow implementation through a regional (mini-) collaborative model.

**Stratification**: For baseline case-fatality, we split the 60 hospitals into 3 strata: ‘low’ (under 5%, n = 6), ‘high’ (5 to 26%, n = 5) and ‘unknown’ (all private hospitals, n = 49). For location, we split the districts into four further strata in each state. For labour room admissions, we split the hospitals into under 1000 in a 3-month period (n = 6); over 1000 (n = 5); and ‘unknown’ (n = 49).

**Figure 2.**
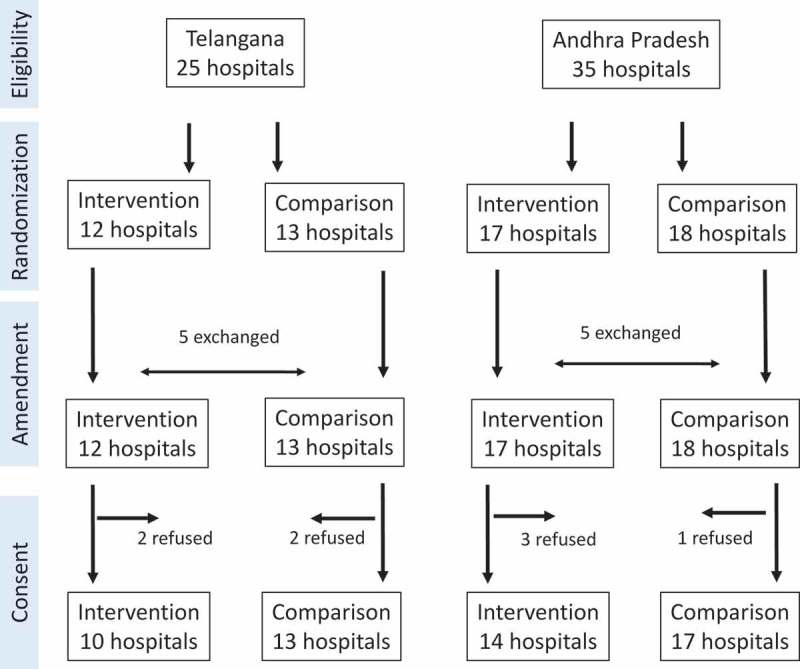
Trial flowchart.

**Allocation**: We listed the 60 hospitals in order of baseline neonatal mortality group, and within that district group, and within that admission group. We then split this list into 30 ‘pairs’ of hospitals. We then took a random number of either 0 or 1 to allocate one of each ‘pair’ of hospitals to the intervention group. This is equivalent to 30 tosses of a coin, and has 2**30 = 1,074 million realizations, so is highly unconstrained. We then adjusted for regional (mini-) collaborative model and exchanged five hospitals in each state to comply with the programmatic decision to include regional hospitals learning from each other, creating a non-randomized study (Figure 2).

### Case study selection

Four case studies were purposely selected among wave II hospitals in Telangana, based on their engagement in the programme at mid-point during implementation, including diversity in hospital types (public/private, and teaching status), admission load and attitudes towards quality improvement. Moreover, a case study of a mini-collaborative was conducted to gain insights about the emerging referral-based mini-collaborative model.

### Analysis

We will tabulate our indicators of implementation of EBP, stillbirth and neonatal mortality, stratified by state, facility type (private or public, college), caseload, type and level of neonatal care unit and implementation strength. A difference-in-difference approach will be used to assess changes between baseline and endline for indicators of processes reflecting 20 EBP as well as neonatal mortality and stillbirth rates [44]. We will adjust for the clustering at hospital level. All analysis will be done in Stata version 13. The plausibility of findings will be reviewed using data on context and implementation strength in facilities.

The cross-sectional study exploring the association between contextual factors and uptake of the EBP will aim to develop a score for readiness as a composite of five contextual factors identified in the literature as being related to organizational readiness for change [45] and the uptake of EBP in facility settings of low- and middle-income countries [46], and consistent with a framework to understand success of quality improvement initiatives [47]. We will test whether the readiness scores change over time and explore their correlation with cluster level summaries for each outcome of interest by hospital newborn care units and labour ward, separately, using Spearman’s rank correlation and a regression model, if appropriate.

Qualitative data will be coded and analysed thematically using NViVO. Analysis will aim to identify key themes under each area of enquiry, and their relationships, and will progress iteratively until saturation is achieved [48]. Individual case analysis (triangulating all available data for that case study) will be developed, then findings compared and contrasted across cases [49].

### Dissemination of findings

At a local level, results will be shared with the hospital leadership using lay versions and briefs. The results will be presented during meetings with policy- and decision-makers of the insurance schemes, and regional meetings on improving quality of perinatal care.

Presentations are planned for relevant national and international conferences such as Health Systems Global, the Forum on quality and safety, the conference of the International Federation of Gynaecology and Obstetrics (FIGO), Evidence-based Neonatology and others. We will prepare a manuscript showing the results of the intervention on stillbirths and neonatal mortality and on implementation of the EBP. We will prepare additional manuscripts outlining the results of the process evaluation and on the potential of scaling-up this approach.

### Ethics

Ethical approval is granted from LSHTM (LSHTM Ethics Ref: 10358) and PHFI’s Institutional Ethics Committee (IIPHH/TRCIEC/064/2015). The study complies with the International Ethical Guidelines for Biomedical Research Involving Human Subjects and the principles of the declaration of Helsinki [50]. An information sheet is read out to each participant. Consent is obtained from each participating hospital, health provider and mother. Participants can withdraw at any time. Confidentiality is assured, as per institutional guidelines of both involved institutions.

## Discussion

### Innovation and potential impact

To the best of our knowledge, this is the first QIC to target neonatal health in secondary and tertiary hospitals in a middle-income country, linked to health insurance scheme.

Our evaluation covers impact, outcome and output indicators, which allows the assessment of changes at several stages along the implementation pathway (Web annex B). With this approach we provide evidence for learning beyond the effect of the intervention.

Tertiary and secondary hospitals are complex entities, often hierarchically organized, where changes in practices demand engagement spanning from hospital leadership, administration and financing, to the ward leadership and individual health providers. As such, these hospitals can be seen as complex adaptive systems [51,52]. The complexity of the Safe Care, Saving Lives quality improvement strategy is also characterized by the fact that both public and private hospitals are included, and that two health insurance schemes are engaged as an umbrella organization. This provides a unique opportunity to analyse the role of context in quality improvement. While many contextual factors have been identified as important for successful quality improvement [47,53], and a few have been tested empirically [54], evidence is largely from high-income settings, and with mixed results [55]. Contextual factors affecting EBP in low- and middle-income settings have been explored and a survey tool developed [46], but this has not been widely applied empirically yet, and never to a quality improvement intervention.

Our process evaluation is theory driven and will refine hypotheses about how a structured quality improvement approach may contribute to institutionalization of EBP, and the extent to which inter-hospital collaboration can add value in this context. The process evaluation will contribute to the growing evidence body on mechanisms of change in relation to quality improvement [56,57], and will question assumptions about the engagement of health workers and hospital leaders in quality improvement in a middle-income country setting, at a time when the scale- up of structured quality improvement is internationally advocated to accelerate reductions in newborn mortality [58].

Exploring the interaction between the intervention and its health system, the evaluation will test the programme assumption that institutionalization of quality improvement in both private and public hospitals can be promoted through external levers, and assess the potential of government-sponsored health insurance schemes in promoting and sustaining quality improvement.

While most of the improvement topics do not demand major investments, active teams improving the quality of care are likely to find that there are limits to their ability to improve. Our process evaluation is expected to show such limitations [17], and document the challenges of embedding quality improvement into the health systems.

### Methodological considerations

Although we originally planned to use a randomized study design for the impact evaluation, this was not feasible due to the programmatic decision to enrol hospitals to allow the formation of regional mini-collaboratives around referral networks at short notice. Nevertheless, the non-randomized allocation achieved a good balance with respect to key hospital characteristics, such as the distribution of public and private facilities, caseload and baseline neonatal mortality.

Teams new to quality improvement need time to understand the approach and its opportunities. Time is needed to engage the leadership on quality improvement [59] and mobilize resources [51]. While ideally the main strategies might have been tested and matured during the wave I implementation, the change towards a regional (mini-) collaborative model, and the addition of programme activities with health insurance trusts and health authorities, gave wave II some aspects of a piloting and testing phase. This is likely to lead to delays in implementation which in turn may make changes too small to be detected.

Contextual factors such as state investments into maternal and neonatal care are likely to influence the results of quality improvement in hospitals. We will document changes within the evaluation period as recommended [60] and will review and discuss the results of our evaluation against this background.

Finally, evaluating neonatal mortality in hospitals is challenging because very sick babies are often referred, and some parents with very sick newborns leave against medical advice, if they do not see any chance of survival. Our baseline report suggests that babies discharged, those who left against medical advice and those who were referred have different risk profiles, with babies discharged against medical advice having the highest risk to die [59]. Adjustment for differing risk is needed before comparisons are made on overall hospital-based mortality. Without considering different risk groups hospital-based mortality is inherently biased towards higher mortality in hospitals caring for sicker babies [61]. We will use telephonic interviews with families of discharged babies to overcome the challenge that many neonatal deaths are missed in hospital records because they happen after referral or after leaving against medical advice. However, due to financial constraints we expect to follow only about 90 cases per hospital with telephonic interviews, giving approximately 50% power to be able to indicate a 20% improvement in neonatal mortality.

We included the overall stillbirth rate as an indicator of improvements in the labour ward, in the absence of documentation of intrapartum or fresh stillbirths. However, as antenatal interventions also have an effect on this indicator, careful interpretation of the results will be needed.

While we intend to perform stratified analysis in relation to State, hospital ownership and other characteristics, such as caseload, the power to indicate any changes is limited because of the limited number of clusters and observations per cluster.

We believe our comprehensive evaluation using qualitative and quantitative methods will provide important information on the functioning and effects of quality improvement in the challenging environment of private and public facilities, including colleges and specialized hospitals.

### Trial status

The trial is recruiting and ongoing.

## Paper context

While collaborative quality improvement is globally increasingly employed to improve quality of care and the implementation of essential evidence-based practices, evidence is limited to what extent this approach improves practices and reduces mortality. Our trial aims to add to the limited evidence and also aims to describe the mechanism of change and the context which may or may not support the collaborative quality improvement to impact on the implementation of evidence-based practices.

## Supplementary material

Supplemenatal data for this article can be accessed here.
