# Supplementary material for: Evaluation of the Safe Care, Saving Lives (SCSL) quality improvement collaborative for neonatal health in Telangana and Andhra Pradesh, India: a study protocol
Source: Glob Health Action. 2019 Mar 8;12(1):1581466. doi: 10.1080/16549716.2019.1581466 (PMC6419630; doi:10.1080/16549716.2019.1581466)
Supplement: Supplemental Material [file ZGHA_A_1581466_SM0324.zip › Annex file A.docx]

Web annex A: Health and socio-economic indicators of the study area

|  | India | Andhra Pradesh | Telangana |
| --- | --- | --- | --- |
| Health Indicators^1^ | | | |
| Maternal mortality ratio (2011-13) ^1^ | 167 | 92 | - |
| Infant mortality rate (2015) ^2^ | 34 | 34 | 31 |
| Still birth rate (2015) ^3^ | 4 | 3 | 1 |
| Neonatal mortality rate (2015) ^3^ | 25 | 24 | 17 |
| Early neonatal mortality rate (2015) ^3^ | 19 | 20 | 14 |
| Socio-economic indicators (2011)^i^ | | | |
| Sex ratio (females per 1000 males) ^4^ | 940 | 992 | |
| Literacy rate ^5^ | 74.04 | 67.66 | |
| Female literacy rate ^5^ | 65.46 | 59.74 | |
| Percentage population below poverty line ^6^ | 29.8 | 21.1 | |

**^I^***Available for Andhra Pradesh before bifurcation of State into Andhra Pradesh and Telangana*

^1^ Sample Registration System. Registrar General of India. *SRS Estimates of Maternal Mortality* Ratio, *2011-13* [cited 2017 25^th^ Oct]; Available from: <http://www.censusindia.gov.in/vital_statistics/mmr_bulletin_2011-13.pdf>

^2^ Sample Registration System. Registrar General of India. *Estimated Birth rate, Death rate, Natural growth rate and Infant mortality rate, 2016* [cited 2017 25^th^ Oct]; Available from: <http://www.censusindia.gov.in/vital_statistics/SRS_Bulletins/SRS%20Bulletin%20-Sep_2017-Rate-2016.pdf>

^3^ Sample Registration System. Registrar General of India. *Chapter 4- Estimates of mortality indicators, 2015* [cited 2017 27^th^ September]; Available from: <http://www.censusindia.gov.in/vital_statistics/SRS_Report_2015/8.Chap%204-Mortality%20Indicators-2015.pdf>.

^4^ Census of India. Registrar General of India. *Chapter 5- Gender Composition, 2011* [cited 2017 27^th^ September]; Available from: <http://censusindia.gov.in/2011-prov-results/data_files/mp/06Gender%20Composition.pdf>

^5^ Census of India. Registrar General of India. *Chapter 6-Literacy, 2011* [cited 2017 27^th^ September]; Available from: <http://censusindia.gov.in/2011-prov-results/data_files/india/Final_PPT_2011_chapter6.pdf>.

^6^ Government of India, New Delhi. *Chapter 2-Socio-economic indicators, National Health Profile, 2011.* [cited 2017 27^th^ September]; Available from: <http://cbhidghs.nic.in/writereaddata/mainlinkFile/07%20Socio-%20Economic%20Indicators%20%202011.pdf>
