# Supplementary material for: Evaluation of the Safe Care, Saving Lives (SCSL) quality improvement collaborative for neonatal health in Telangana and Andhra Pradesh, India: a study protocol
Source: Glob Health Action. 2019 Mar 8;12(1):1581466. doi: 10.1080/16549716.2019.1581466 (PMC6419630; doi:10.1080/16549716.2019.1581466)
Supplement: Supplemental Material [file ZGHA_A_1581466_SM0324.zip › Annex file B.docx]

**Webannex B - Safe Care Saving Lives Implementation strategy and theory of change**

**Contents**

1. Background 1
2. Improvement work – hospital level 3
3. Support structure and delivery 11
4. Collaborative platform 14
5. Intervention at the health system level 15
6. Programme theory of change 16

5.1 Changes within each hospital 16

5.2 Changes at Quality Improvement collaborative level 18

5.3. Changes at health system level 20

References 22

**___________________________________________________________________________**

1. **Background**

***Programme design and partnership arrangements***

The Safe Care Saving Lives programme intervention was designed by Access Health International (hereafter ACCESS) in partnership with the Institute for Health Care improvement, modelled on the Project *5 Alive* in Ghana ([2](#_ENREF_2)).

The programme was designed to respond to the need to improve quality of care and processes in special newborn care units, identified through an assessment of fourteen newborn care units in the former state of Andhra Pradesh, undertaken by the National Rural Health Mission Andhra Pradesh, UNICEF and the Commissioner Health and Family Welfare in 2011-12, with participation from ACCESS. The programme was designed following a previous collaborative quality improvement experience: in August 2014, ACCESS Health India, together with the Institute of Healthcare Improvement, brought together representatives from six of the best public and private neonatal intensive care units in India to develop and pilot a standard set of hospital processes to reduce the number of care driven infections among newborns. In this early “Indian Neonatal Collaborative”, practitioners worked together to learn, implement, and scale up practices that reduce newborn deaths caused by infection (Unpublished).

The Safe Care Saving Lives programme was developed in partnership with the Rajiv Aarogyasri Health Care Trust. The Aarogyasri Health Insurance scheme is a unique community health insurance scheme that provides financial protection to families living below the poverty line for the treatment of serious ailments requiring hospitalization and surgery, including treatment of small and sick newborns. The scheme reimburses empanelled hospitals for the provision of services to eligible groups, and hospitals must meet six empanelment criteria relating to infrastructural setting in order to benefit from the scheme ([3](#_ENREF_3)).

ACCESS signed a memorandum of understanding with the Rajiv Aarogyasri Health Care Trust in June 2014 with the aim to reduce perinatal and neonatal mortality by fifteen percent over a four-year period (2014-2018) in the 85 public and private sector neonatal care units empanelled with the Rajiv Aarogyasri Health Care Trust. Following the bifurcation of the former state of Andhra Pradesh in 2014, the Rajiv Aarogyasri Health Care Trust was split into the Aarogyasri Health Care Trust in Telangana and the Dr. Nandamuri Taraka Rama Rao (NTR) Vaidya Seva Trust in Andhra Pradesh in August 2015. This triggered the need to renegotiate partnership arrangements in the two states.

All public health facilities are directly managed and governed by the office of the Commissioner Health and Family Welfare and are supported under the national health mission, so a tripartite memorandum of understanding involving Aarogyasri Health Care Trust, ACCESS Health International, and the Commissioner Health and Family Welfare was signed in January 2017 in Telangana. In Andhra Pradesh, a memorandum of understanding was being developed at the time of writing.

***Programme timeline and overview***

Implementation was designed in 3 waves or phases including 85 hospitals in two states, jointly identified by Aarogyasri Health Care Trust (before the split) and ACCESS Health International:

- Wave I (2014 – 2016) involved two components: the identification of focus evidence-based practices by an Expert Faculty Group and development of the Quality Improvement Toolkit for participating hospitals (details under section 1 below), followed by implementation in 25 hospitals that volunteered to participate in the intervention. Hospitals were invited to attend a high-level project launch in August 2014, where leading clinicians in the field of Neonatology and Paediatrics from the Safe Care Saving Lives Expert Faculty Group presented evidence on the main drivers of newborn mortality and morbidity and potential improvements in care practices and processes to address them. Participating hospitals were also introduced to the quality improvement approach through success stories and invited to apply to participate in the Safe Care Saving Lives programme. The 25 initial hospitals were selected among 29 applicants following visits from the programme team to discuss the hospitals’ motivation and review available care facilities based, on a self-assessment form ([4](#_ENREF_4)). Wave I was a phase of programme refinement and adaptation, as is typical in phased implementation of quality improvement programmes ([5](#_ENREF_5)). In 2016, the programme design was substantially revised to incorporate lessons learned and to allow renegotiation of institutional partnership agreements after the state bifurcation.
- Wave II, originally planned to start in December 2015, began in February 2017, involving 29 of the remaining 60 eligible hospitals, identified in partnership with State governments. However, two of the identified hospitals refused to participate, thus the intervention initiated in only 27. Wave II is the focus of this paper.
- Given delays on the original timetable for implementation, wave III, originally planned for 2017 and involving 31 hospitals, was planned for the second half of 2018.

The Safe Care Saving Lives initiative was based on the collaborative quality improvement approach developed by the Institute for Health Care Improvement in the Breakthrough Series Model ([6](#_ENREF_6)). The approach is called a “collaborative” because teams from several hospitals work together in a structured way to improve a specific practice related to newborn care, and is defined by the following features ([6-9](#_ENREF_6)):

1. A focused clinical subject (evidence-based practices for newborn mortality reduction)
2. Learning from experts in field of Obstetrics, Neonatology and Quality Improvement
3. Multi-professional teams from multiple hospitals participate
4. Teams use a structured approach for quality improvement (setting targets, collecting data and testing changes).
5. A series of learning sessions between hospitals

In its second wave (wave II), the programme was implemented at three interconnected levels:

1. At the level of individual participating hospitals, where hospitals implemented quality improvement activities in newborn care units and, if available, labour rooms (for details on hospital recruitment, see study population and randomisation sections of the protocol paper).
2. At the collaborative level, where groups of hospitals shared learning and experience of quality improvement
3. At the state health system level, where the programme engaged institutional stakeholders to promote and prioritise quality improvement.

The implementation strategy at each of these levels is described in detail below, followed by the programme support structure and theory of change. Although the programme approach at its core entailed a collaboration between different facilities, the implementation strategy is described starting from the individual hospital level, because this best represents the hospital engagement approach used by ACCESS Health International in wave II. While the original Breakthrough Collaborative model and the model used in wave I were top down (hospitals joining a collaborative, attending an initial group learning session and then beginning their own quality improvement activities), the approach used in wave II was bottom-up (hospitals engaged in quality improvement individually and then linked in collaborative efforts). Also the system-level component was added after the wave I review, to complement and aid feasibility, effectiveness and sustainability of direct and collaborative quality improvement activities.

**2. Improvement work – hospital level**

**The Model for Improvement approach**

The intervention quality improvement approach is based on health facility teams working towards improved adherence to evidence-based practices (EBPs), that if optimally implemented have the potential to reduce newborn mortality and stillbirths. These practices targeted key drivers of newborn mortality and stillbirths (namely: complications of prematurity; newborn sepsis and birth asphyxia) through interventions during intra-partum and the early newborn care period. These were identified in early 2014 by an Expert Faculty Group, including over 35 local, national and international technical and clinical experts, and were collated into a guide for participating hospitals, referred to as a “Safe Care Saving Lives Quality Improvement Toolkit”([10](#_ENREF_10)). This Toolkit described the focus evidence-based practices, measurement indicators and audit tools, and included possible change ideas to test, based on successful experience elsewhere. The Safe Care Saving Lives Quality Improvement Toolkit was revisited at the end of wave I. The original Safe Care Saving Lives Quality Improvement Toolkit used in wave I hospitals detailed 15 evidence-based practices; another six practices were added and 1 dropped in March 2017, incorporating new evidence and experiences in implementing and testing the changes. The Safe Care Saving Lives Quality Improvement Toolkit used in wave II included 20 EBPs, organised in “bundles”, each of which was a combination of two or more EBPs to address a focus area such as sepsis, prematurity and birth asphyxia.

Table 1 provides an overview of the evidence-based practices targeted by the intervention.

**Table 1 – Description of evidence-based practices by bundle**

|  | **Sepsis bundle** | **Prematurity bundle** | **Asphyxia bundle** |
| --- | --- | --- | --- |
| Practices promoted in labour rooms | - Antibiotics to women at risk of sepsis - Hand hygiene & gloves during per-vaginal examination - WHO 6 cleans | - Ante-natal steroids - Early breastfeeding | - High risk categorization of woman in labour - Trained personnel for high risk delivery - Compliance with partogram - Pre-delivery checklist - Compliance with oxytocin infusion protocol - Resuscitation with bag and mask |
| Practices promoted in neonatal care units | - Hand hygiene - Rational usage of antibiotics - Intravenous tubing - Protocol for central vascular catheter - Aseptic Peripheral IV line insertion | - First temperature in 15 minutes from admission - Exclusive breastfeeding - Kangaroo Mother Care | - CPAP in preterm neonates with respiratory distress |
| **Total no. practices** | **8** | **5** | **7** |


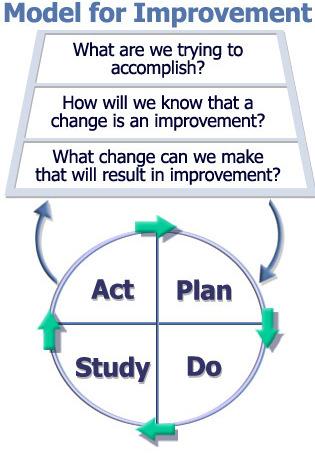
The intervention involved the formation of Quality Improvement (QI) teams in labour / delivery rooms and Special Newborn Care Units / Neonatal Intensive Care Units of participating hospitals. QI teams were formed of health care workers from the target units, supported by ACCESS staff (see section 2 below) to implement quality improvement activities, and develop a problem-solving approach towards the adoption of selected evidence-based practices.

***Figure 1. Model for Improvement (***[***1***](#_ENREF_1)***)***

The programme used the continuous quality improvement approach developed by Langley et al. and adapted from the automobile industry to health care organisations, known as the “Model for Improvement”, shown in Figure 1 ([1](#_ENREF_1)). The approach hinges on a cycle of setting an aim for improvement; agreeing progress measures; identifying a suitable innovation (change); and continuous testing and refinement of changes through collection and review of data, to measure progress towards the desired aim, in cycles known as Plan-Do-Study-Act ([1](#_ENREF_1), [6](#_ENREF_6)).

The Model for Improvement shares with other quality improvement approaches the focus on continuous quality improvement, but it has a distinctive theoretical underpinning in Deming’s system of profound knowledge, or the interplay of theories of systems, knowledge, variation and psychology ([1](#_ENREF_1)).

- In line with systems theory, the approach assumes interdependence in a system, and therefore emphasises the monitoring of unintended consequences of change through so-called “balancing measures”. Importantly, the approach also posits that “every system is perfectly designed to deliver the results it produces” ([1](#_ENREF_1))(p.79), therefore fundamental change is required to improve performance in the long-term.
- The approach emphasises that knowledge is built through an iterative deductive and inductive approach. The identification of change ideas (Plan phase) is based on a prediction on the effect of the introduction of the change. The better the knowledge of the system in which the change is being introduced, the more accurate the prediction can be. The planning phase is essentially about articulating a theory about how change will happen, and putting it to the test through the Do phase (deductive knowledge building). Once the test on a small scale is completed, in the Study and Act phases, data is used to either refine, discard or adopt the idea, and refine hypotheses about the system’s response to change (inductive knowledge building) (6).
- The approach is data-driven, and relies on longitudinal analysis to detect variation over time. Quality improvement efforts are supported by collection of data against relevant measures, and these are regularly plotted in run-charts, and analysed to estimate the effect of introduced changes, and to determine the necessary course of action.
- The approach emphasises that specific strategies altering permanent support structures are required to promote the implementation (adoption) of a change idea that has proven effective in the testing phase, for example redesigning job descriptions, or providing in-service training systematically. This will mean that the change gradually involves larger groups of people, hence the need to focus on both individual and collective psychology when introducing changes. The approach draws from theories of behaviour change, and stresses the importance of understanding team members’ intrinsic and extrinsic motivation, and of anticipating and dealing with resistance with appropriate communication strategies.

It is important to note that, although this quality improvement approach has explicit key principles, one of its distinctive feature is adaptability. The Model for Improvement is rather a problem-solving approach, a mindset more than a set of tools ([11](#_ENREF_11)).

The use of the Model for Improvement in a collaborative programme also has another important distinctive feature. In a continuous quality improvement approach used in individual health settings, teams choose their own issues of focus, and spend time identifying problems, causes, and solutions, which may or may not draw on evidence about effective interventions in the focus area ([6](#_ENREF_6)). In a collaborative approach, this evidence is already condensed in the “Safe Care Saving Lives Quality Improvement Toolkit”, and topics for improvement are identified from a pre-identified set of practices that are known to reduce a specific driver of newborn mortality. As a result, the diagnosis time is shortened, and hospitals are encouraged, though not obliged, to test innovation and ideas that have already proven effective in a similar context ([7](#_ENREF_7), [12](#_ENREF_12)).

**Engagement of participating hospitals**

Prior to starting implementation of improvement activities, ACCESS staff (mentors) raised awareness of the hospital leadership on the importance and opportunities for quality improvement in newborn care, and actively sought their engagement and buy-in to the programme. This was described as a “sensitisation” phase, consisting of multiple face-to-face visits and communications, primarily with the Medical Superintendent and Head of Departments (newborn care unit and Labour Room). In this phase, the hospital leadership was introduced to the programme, the expected outcomes, and given an overview of the quality improvement approach. This phase lasted 1 – 4 months, or longer, depending on the leadership’s interest and response.

**Quality Improvement teams and their composition**

Following a positive response from hospital leadership, mentors facilitated the formation QI teams in the target departments, generally 2 teams per hospital. Doctors, nurses, and data entry operator were invited for the first orientation / sensitization session, during which the department leadership nominates specific health workers to be part of the QI Team, based on their involvement in the newborn care unit or labour room, and his or her interest in the quality improvement initiative.

The teams generally comprised 4-5 clinical and administrative staff from each Department. These could include: 2 doctors (Paediatrician/Obstetricians), 1-2 resident doctors (in case of teaching hospitals) and 2-3 nurses per department. Where feasible, the Head of Hospital Administration and a data entry operator from the newborn care unit were also included. The structure of the QI teams was not fixed, but flexible depending on the availability and interest of the staff.

**Quality improvement activities implemented by Quality Improvement teams**

***Gap analysis***

ACCESS mentors conducted gap analysis using two checklists (for labour room and SNCU) to assess infrastructure, equipment, human resources, protocols and processes, and records maintenance. They summarised the identified gaps, and the level at which these could be solved. Access mentors shared the results with the hospital leadership and discussed major gaps and clinical priorities. ACCESS mentors worked with the hospital QI team on further problem analysis and identification of priority areas.

***Identification of priority areas of focus***

Mentors facilitated the initial baseline data collection from clinical data in order to build a historical trend on admission rates, newborn mortality, and respective causes, based on the previous 12 months. Mentors undertook the initial analysis and presented it back to QI teams, as well as provided an initial introduction to the Model for Improvement for the QI teams. Based on this, a priority issue for improvement was selected among the main drivers of mortality (sepsis, prematurity or asphyxia), and 1 or 2 EBPs contributing to reduction of that driver of mortality are prioritised per hospital. Targets for improvement were set at >80% coverage, based on the expectation that coverage of EBPs at scale be required to impact on mortality.

Once the focus area (sepsis, asphyxia, prematurity) was identified and the team had decided on which EBPs they wanted to start, mentors provided training and coaching to QI team members to use the Model for Improvement approach. They facilitated QI teams’ analysis of causes of poor adherence to the focus practice, using tools such as process mapping, root cause analysis and bottleneck analysis.

***Generation of change ideas***

The QI team brainstormed on “change ideas”, or innovative practices that could potentially improve the adherence to the identified evidence-based practice. Change ideas were identified by the team themselves or the team used one of the change ideas already used in other hospitals. Based on the learning from the quality improvement work in wave I, ACCESS developed a change package which included a list of change ideas that were tested and adopted. As explained above, the rationale for introducing change ideas selected from the “change package” was that it may shorten the time required by teams to identify solutions, promoting diffusion of innovation among hospitals, and therefore accelerating the achievement of results.

Table 2 describes examples of change ideas relevant to each EBP.

**Table 2: Change ideas for evidence-based practices**

| **EBP** | **Change idea** |
| --- | --- |
| **MANAGEMENT OF SEPSIS** | |
| Ensure appropriate use of Antibiotics | - Follow a unit level policy on antibiotic use - Adopt the All India Institute of Medical Sciences sepsis management algorithm for antibiotic administration - Every SNCU/NICU must have its own antibiotic policy based on the culture and antibiotic sensitivity report from a reliable laboratory - Multidisciplinary team - Assess the baseline adherence to appropriate antibiotic administration - Audit of case sheets/drug consumption - Ensure supply of antibiotics is in consonance with the unit’s antibiotic policy - Antibiotic stewardship – permission of consultant if deviations from policy are necessary |
| Ensure Per Vaginal examination is conducted correctly in the Labor room | - Ensure continuous availability of running water and gloves of all sizes - Train all the labor room staff on sterile Per Vaginal examination - Assign responsibility to specific nurses to perform the Per Vaginal examination - Limit the number of Per Vaginal examinations to three for women in labor - Display ‘Steps of sterile Per Vaginal examination’ poster in the Labor Room |
| WHO 6 cleans | - Protocol dissemination - Audit |
| Ensure compliance with optimal hand hygiene practices among all staff in the SNCU / NICU | - Install elbow operated taps in front of the NICU AND Install hand dryers to wipe wet hands - Introduce alcohol based hand rub at every bed - Reorient and train the staff on correct steps for hand washing - Use autoclaved newspaper to wipe wet hands - Keep 'Hand washing register' at the entry of NICU - Stick 'Hand wash reminder' poster on warmer - Floor taping in red color at the entrance of newborn area - Conduct regular audits using video recordings at NICU |
| Ensure compliance to Aseptic Non Touch Technique (ANTT) during Peripheral Intravenous line insertion | - Delegate two nurses to perform the intravenous cannulation procedure - Keep a dedicated autoclaved trolley set for intravenous Cannulation procedure - Ensure adequate availability of peripheral intravenous insertion kits in the crash carts. - Sensitize the staff - Modify step four of Aseptic Non Touch Technique from cleaning of the trolley surface to spreading the autoclaved cloth of sterile set on trolley surface - Flip the cover of the kidney tray to place the equipment used for intravenous cannulation. - Display 'Nine steps of Aseptic Non Touch Technique' poster in the unit - Conduct of cross audit by the medical officer apart from nurses |
| Antibiotics to neonates born to mother with risk factors for sepsis | - Protocol dissemination - Audit |
| Prevent ventilator associated Pneumonia | - Protocol dissemination - Audit |
| **MANAGEMENT OF PREMATURITY COMPLICATION** | |
| Ante-natal steroids | - New format to capture details of administration of Ante-natal corticosteroids - Use ultrasound scan to assess the gestational age of pregnant women arriving with complaints of labor pains - Protocol dissemination - Audit |
| Early breastfeeding  Exclusive breastfeeding  Counselling on expressing breast milk | - New format for recording Expressed Breast Milk - Ensure counseling is given properly - Distribute the work among the team instead of relying on one person. - Protocol dissemination - Audit - Ensure privacy - Demonstration (milk expression and feeding) |
| Take the first temperature of all babies in the Neonatal Care Units within fifteen minutes of admission | - New format for recording the baby’s temperature - Designated triage in the Casualty - Monitor the temperature of the Labor Room - Record temperature and time of measurement of the newborn inside Labor Room on the case sheet - Leave warmer switched on for ten to fifteen minutes before shifting the baby - Note first temperature within fifteen minutes in the designated triage area of Neonatal Intensive Care Unit - Record temperature at first point of contact-ambulance/casualty. Note the referral point and mode of transportation of babies coming to Neonatal Intensive-Care Unit in outborn cases; Use transport incubator - Ensure correct temperature in preparation for resuscitation/before transport - Cover baby - Protocol dissemination - Audit |
| Continuous Positive Airway Pressure (CPAP) therapy for Respiratory Distress Syndrome | - Policy for early initiation of CPAP (preferably in the labor room) and continuation of the same (depending on the clinical indications) - Ensure availability of CPAP with tubings - Protocol for referral of babies who have deteriorated on CPAP/for those who need advanced ventilation. - Protocol dissemination - Audit |
| Kangaroo Mother Care | - Protocol dissemination - Ensure privacy of mother by creating a dedicated clean and private area Provision of gowns or KMC bags in the unit - Proper demonstration of the correct method of KMC - Videos & Posters as reminders - Frequent assessment of the knowledge and skills of the care givers (e.g. nursing staff) pertaining to KMC - Encourage mothers to interact/counsel one another - Encouraging fathers to provide KMC in the hospital - Establish dedicated phone line at the facility for answering the queries of the mothers post discharge - Home visits by the nursing staff/ care givers to ensure the practice of domiciliary KMC |
| **Management of Birth Asphyxia** | |
| High risk categorization of woman in labour | - Protocol dissemination - Audit - Use Government of India, Dakshata, Maternal and Newborn Health checklist for categorizing high risk cases. - Sensitize Labor Room nurses on categorization of high risk mothers - Use a "flower" sign / High risk stamp / 'High risk' label with red pen to mark all the high risk women case sheets. - Reorient the staff periodically on Maternal and Fetal risk factors - Write the high risk cases on a black board inside the Obstetrics & Gynaecology department. - Arrange an alarm bell in Labor Room & Operation Theatre to ‘connect’ Sick Newborn Care Unit. - Establish a communication linkage between both Obs (Labor Room) and Sick Newborn Care Unit (Pediatrics). Use a mobile phone to call the Duty medical officer. - Track the outcome of women till delivery, to determine if delivery is normal or Caesarean section, and the condition of baby - Keep separate register for noting High risk cases with their condition. - Put up a chart to capture data on High risk Cases on a daily basis. - Stick high risk conditions poster in the Labor Room for reminding the nurses |
| Compliance with partogram | - Protocol dissemination - Audit - Ensure availability of partograph sheets in the Labor Room. - Conduct a training session for post graduate trainees on filling/documenting partograph. - Delegate training responsibility to two Skilled Birth Assistants to train the other staff. - Train all nurses on partograph sheet at the beginning of every month. - Periodic retraining schedule for the new staff on rotation to Labor Room. - Fill the essential components of partograph - Attach the Partograph sheet within the case sheet to ensure it is filled during the course of labor - Post Dakshata trained staff nurses in the morning and afternoon shift. - Post at least one nurse trained in Skilled Birth Assistance in each shift or post senior most staff on duty. - Create a WhatsApp group with the staff to ensure that partograph data is captured real time. - Include the partograph data in the daily duty statistics of Labor Room Medical Officer register. - Ensure that a gynecologist is present at all times and that surprise visits are conducted by the Head of Department. - Ensure partograph sheet is filled by duty Doctors. - Monthly review by Head of the unit. |
| Compliance with oxytocin infusion protocol | - Protocol dissemination - Training - Audit |
| Trained personnel for high risk delivery | - Protocol dissemination - Audit - Stick the contact details of the Duty Medical Officer in NICU. Use mobile for calling. - Ensure that all resuscitation equipment are available when the SNCU staff attend the case. - Retrain all the SNCU staff in Newborn Resuscitation Protocol protocols if sufficient staff available, else, train Labor Room staff. - Train more staff in Newborn Resuscitation Protocol. - Swap one staff between the Gynecology and Pediatrics department - Nurses trained in Newborn Resuscitation Protocol are resourceful during high risk deliveries. - Swap the staff for a limited period of a time for on job training of Labor Room staff. - Post SNCU staff in labor wards on a rotation basis. - Fix Labor Room postings of Newborn Resuscitation Protocol trained staff. - Note the time difference between call by nurse and arrival of pediatrician in high risk register. - Capture the number of high risk cases attended in the pediatric department. |
| Pre-delivery checklist | - Checklist adaptation/tailoring - Training - Test preferred mode of checklist dissemination (paper-based, laminated, visual in labour room) - Audit availability of items in the checklist - Audit checklist use |
| Resuscitation with bag and mask | - Protocol dissemination - Training - Improve team work through teamwork assessment scales |

***Use of Plan-Do-Study-Act cycles***

QI teams were supported to use a structured cycle for quality improvement activities, or Plan-Do-Study-Act (PDSA) cycle, (as suggested by the Model for Improvement outlined above), to understand whether change ideas result in improvement. Each cycle includes four stages:

*Plan* - planning the introduction of a change idea in the QI team, and gaining support for it from the head of Department and hospital leadership.

*Do* – implementing the change. Specific QI team members were tasked with the introduction of the change idea, and other clinical staff from the Department could be asked to support this improvement work on an ad hoc basis.

*Study* – regular audit and review of results to study whether the change had resulted in improvement in a given context. This was led by members of the QI team, and involved tracking selected indicators on a regular basis (daily / weekly/ fortnightly).

*Act*–taking a decision on the basis of the “study” results. This could include adoption of the change idea, i.e. a decision to continue with it and integrate it in clinical practice; adaptation of the change idea, for example modifying some aspects to ensure a better fit with the hospital setting; discard or abandon the change idea.

It should be noted that the approach was not necessarily sequential, but was logically structured. For example, the cycle could start from the Study component, or the planning phase could be shortened if change ideas are simple. Decisions (under the Act part of the cycle) could entail further testing (adaptation) in which case the cycle was repeated. Multiple cycles were generally required, both to refine and adapt the change idea to the individual setting, and to ensure change ideas build on each other, and over time to address the multiple root causes identified.

Mentors coached QI teams to use the Model for Improvement approach, without using jargon such as PDSA cycles. The programme aimed for QI teams to become competent in using a structured method for identifying and responding to barriers to the adoption of an EBP.

Table 3 provides an overview of wave II progress in implementing QI activities in the first three quarters of the intervention.

**Table 3 – Implementation description**

|  | **Quarter 1**  **(April – June 2017)** | **Quarter 2**  **(July – Sept 2017)** | **Quarter3**  **(Oct- Dec 2017)** |
| --- | --- | --- | --- |
| **Uptake of EBPs** | | | |
| Total no. EBPs started (across all hospitals) | 17 | 50 | 23 |
| - Sepsis package | 5 | 17 | 4 |
| - Prematurity package | 8 | 24 | 17 |
| - Asphyxia package | 3 | 9 | 2 |
| - Vitamin K administration | 1 | 0 | 0 |
| **Implementation of QI activities** | | | |
| Total no. change ideas tested | 27 | 132 | 52 |
| - Sepsis package | 8 | 56 | 9 |
| - Prematurity package | 12 | 56 | 32 |
| - Asphyxia package | 7 | 20 | 11 |
| **Average no. PDSA cycles used for change idea by package** | | | |
| - Sepsis package | 1.0 (8/8) | 1.2 (68/56) | 1.3 (12/9) |
| - Prematurity package | 1.4 (17/12) | 1.4 (79/56) | 1.1 (36/32) |
| - Asphyxia package | 1.0 (7/7) | 1.0 (20/20) | 1.3 (14/11) |
| **Support to QI teams** | | | |
| Total no. mentor-facility contact | 72 | 142 | 105 |
| Average no. of mentor-facility contact per hospital | 4.2 (72/17) | 5.9 (142/24) | 5.8 (105/18) |
| No. hospitals with at least one quarterly contact with mentor | 15 | 23 | 16 |
| **Focus of mentor-QI team contact** | | | |
| No. hospitals receiving QI-related training | 15 | 21 | 17 |
| No. of hospitals receiving clinical or protocol-related training relevant to EBP of focus | 4 | 11 | 10 |
| No. of hospitals receiving other type of support/facilitation (e.g. equipment or infrastructure related or posters) | 3 | 10 | 10 |
| Remarks | 2 hospitals dropped early not included in this data.  Info not recorded for 10 hospitals.  Info available for N=17 | 2 hospitals dropped early not included in this data.  Info not recorded for 3 hospitals.  Info available for N=24 | 2 hospitals dropped early not included in this data.  Additional 3 hospitals dropped.  Info not recorded for 6 hospitals.  Info available for N=18 |

1. **Support structure and delivery**

ACCESS supported participating hospitals through mentors, who were generally public health professionals specifically recruited for this role, trained on the quality improvement methodology. At the beginning of wave I, nine ACCESS mentors attended a 2-day training on the Model for Improvement approach, delivered by the Institute for Healthcare Improvement (IHI), of which, at the end of 2017 three were working as mentors, other staff having been lost due to attrition. Team members recruited later in the programme life attended online training on the Model for Improvement through the IHI Open School ([13](#_ENREF_13)), complemented by in-house training delivered by the Programme Manager.

Each hospital was supported by a Quality Improvement (QI) Mentor and a Senior Associate or Quality Improvement (QI) Lead. Mentors mostly had 2-5 years experience after post-graduate studies, and generally less than two years of experience in quality improvement. Senior Associates or QI Leads were more experienced mentors, as summarised in table 4 below. A programme organogram is provided in figure 2.

**Table 4 – Overview of ACCESS team**

| **Function** | **Role** | **No. staff** | **No. staff with clinical background** | **No. staff trained in QI** | **No. staff trained in QI by IHI** | **Years of experience (mean)** | **Years of experience in QI (mean)** |
| --- | --- | --- | --- | --- | --- | --- | --- |
| **Hospital mentors** | Quality Associate | 6 | 0 | 5 | 5 | 2-4 (2.5) | ≤ 2 years (1.1) |
|  | Quality Lead/Senior Quality Associate | 6 | 1 | 5 | 3 | 4 - 9.5 (6) | 0.75 - 9.5 (4) |
| **QI Cell staff** | Quality Advisor/Data Analyst | 2 | 1 | 1 | 1 | 12-14 (13) | 0 - 0.5 (0.25) |
| **AHI Management** | Director QPI/Program Manager/M&E Lead | 3 | 2 | 2 | 1 | 9 -15 (12) | 1-5 (2.7) |
| **Other** | Other consultants | 3 | 0 | 2 | 0 | 2.5 - 4 (3) | 0 - 3 (1.8) |

The Quality Improvement (QI) Associate was primarily responsible for capacity building, technical support, coaching and follow up to QI teams, and the senior member (Senior QI Associate or QI Lead) complemented the technical support provided by the QI Associate, and worked with the hospital leadership to facilitate the removal of bottlenecks to quality improvement and the adoption of EBPs. QI Leads were responsible for 1-3 QI Associates who in turn mentored 3-5 hospitals. In addition to supporting activities with individual hospitals, they conducted baseline and regular aggregate data analysis to identify common challenges, and opportunities for collaborative learning. They facilitated learning sessions, with support from the relevant QI Associate, and were responsible for disseminating success stories and lessons learnt.

***Figure 2: Organogram***

 An indicative list of mentors’ activities with participating hospitals is below:

| - Sensitisation and engagement of hospital leadership - Identify and nurture QI champions - Facilitate formation of QI teams - Facilitation of gap analysis and identification of aim for improvement - Capacity building on quality improvement methodology (planning and conducting trainings, sharing knowledge) - Support to ongoing data management and analysis - Mentoring and coaching through regular visits to hospital - Regular contact and follow up with QI teams and leadership (whatsapp, phone) - Support to implementation of change ideas, for example: - Support to resource mobilization (facilitating infrastructural changes) - Facilitation of clinical training (if requested by the QI team as one of their change ideas) - Facilitating internal coordination (e.g. between various cadres; between frontline, physicians and administration; between labour Room and neonatal care units) and externally (e.g. with district health authorities) - Organize, support and follow up from mini-collaborative learning sessions - Disseminate success stories and lessons learnt. |
| --- |

In addition to the facility QI teams and hospital leadership, the programme engaged with the Aarogyasri Health Care Trust, with a QI Advisor and Data Analyst seconded to the Aarogyasri Health Care Trust QI Cell, and ongoing interaction between ACCESS mentors/Leads and the hospital Medco (a medical doctor responsible for the implementation of the Aarogyasri scheme in the facility).

At senior level, the team also included ACCESS Quality and Process Improvement Director, who led on activities at state level and reported to ACCESS headquarters. The programme engaged the formal structure for Quality Assurance under the National Health Mission at state and district level. It comprised of the State Quality Assurance Committee (where ACCESS is represented by the Quality and Process Improvement Director) and District Quality Assurance Committees, where ACCESS – represented by Senior QI Associates – interfaced with District Quality Assurance Managers. ACCESS core team also included a Program Manager and Monitoring and Evaluation Advisor.

1. **Collaborative platform**

In addition to mentoring and coaching of individual hospitals, the “Breakthrough collaborative approach” entails collaborative activities between hospitals working on similar evidence-based practices ([6](#_ENREF_6)). In the Safe Care Saving Lives programme, the quality improvement collaborative was based on the Government-sponsored health insurance platform (i.e. the Aarogyasri Health Care Trust in Telangana and Dr NTR Vaidya Sewa in Andhra Pradesh), engaging hospitals that were empanelled in this scheme and that shared a common aim for improvement of newborn care. Hence, unlike other collaboratives, participation was not voluntary ([6](#_ENREF_6), [12](#_ENREF_12)). The health insurance platform was used to justify the engagement phase (sensitisation) described above, as well as the strategies to promote hospital leadership’s engagement through system-level interventions.

In the Breakthrough Collaborative approach, QI teams from participating hospitals work together over a specific timeframe (often 9-12 months) to learn about quality improvement methods, and exchange ideas and success stories during so-called learning sessions. Participating hospitals usually attend 3-4 face-to-face or virtual sessions over the course of the collaborative timeframe. Between these learning sessions, each hospital introduces and tests changes in their own setting (using the Model for Improvement approach described in sections 1), supported through regular coaching and mentoring.

The Safe Care Saving Lives programme promoted open ended collaboration between participating hospitals, initially facilitating linkages and communication for the duration of the programme implementation. ACCESS organised collaborative learning sessions, by identifying common challenges or priorities for improvement based on baseline and regular data collection; by nurturing champions and QI teams to share their work and be open to learning from other participating hospitals; and by documenting and disseminating success stories and lessons learned through the Aarogyasri Health Care Trust website. The Breakthrough Collaborative model entailing large learning sessions was used in wave I and found to be ineffective given logistic challenges and time requirements to convene large gathering across two States. Therefore, in wave II, the programme formed regional (mini-) collaborative jointly with wave I facilities, mostly consisting of a main referral hospital with its referring facilities. Mini-collaborative learning sessions involved hospitals in each regional collaborative that were working on the same evidence-based practice, and a good performing wave I hospital also participated as a model. These learning sessions could be held face-to-face or virtually. The programme envisaged the health insurance network taking over the facilitation and coordination of collaborative learning sessions at the end of the intervention in each State. Table 5 summarises the implementation of mini-collaborative activities in the first 3 quarters of the wave II.

**Table 5 – Implementation of mini-collaborative activities**

|  | **Quarter 1**  **(April – June 2017)** | **Quarter 2**  **(July – Sept 2017)** | **Quarter3**  **(Oct- Dec 2017)** |
| --- | --- | --- | --- |
| **Support to mini-collaborative** | | | |
| No. mini-collaborative learning sessions conducted | 4 | 3 | 4 |
| No. wave II hospitals attending a mini-collaborative learning session | 4 | 3 | 2 |

**5. Intervention at the health system level**

The Safe Care Saving Lives programme also aimed to strengthen capabilities and the policy framework for scale up of continuous quality improvement at state level. There were two streams of support:

- Support to government sponsored health insurance trusts
- Support to state and district level health authorities

Engagement with the Aarogyasri Health Care Trust originated from the assumption that a health insurance trust would be interested in improving quality of care to reduce costs.

The tripartite agreement between ACCESS, the Aarogyasri Health Care Trust and Ministry of Health and Family Welfare in Telangana, mentioned in the background, committed Aarogyasri Health Care Trust to institutionalising a Quality Improvement (QI) Cell, as a technical and advocacy unit for quality improvement within the Aarogyasri Health Care Trust. The QI Cell was meant to strengthen Aarogyasri Health Care Trust’s capacity for quality control to support empanelled hospitals’ quality improvement efforts. It also provided a data analysis function to stress the case for investment in quality improvement as a strategy for effective and efficient care. ACCESS supported the formation of a QI Cell in Aarogyasri Health Care Trust through the secondment of a Data Analyst and a Quality Improvement (QI) Technical Advisor, on the understanding that these positions will be absorbed into the Aarogyasri Health Care Trust structure at the end of the programme. ACCESS staff in the QI Cell worked towards strengthening Aarogyasri Health Care Trust capacity, and towards developing an incentive system to link Aarogyasri Health Care Trust payments to quality improvement measures. The QI Cell planned to meet monthly, with attendance at senior level in the signatory parties, thus providing a regular quality control function. ACCESS conducted data analysis on claims filed with Aarogyasri Health Care Trust; provided capacity building to Aarogyasri Health Care Trust counterparts for quality improvement mentoring and monitoring; disseminated success stories and lessons learned through the Aarogyasri Health Care Trust website; and provided technical support to the development of the quality improvement incentive package. The support provided to Aarogyasri Health Care Trust focused on building capacity for quality improvement broadly, not restricted to newborn care, and the development of incentives incorporated all the insurance packages of Aarogyasri Health Care Trust, where newborn care is a very small component.

In relation to government health authorities, the programme aimed to build capacity in the State Quality Assurance Committee (SQAC) and district-level Quality Assurance staff. The programme aligned with the National Quality Assurance framework and standards ([14](#_ENREF_14)), and aimed to integrate quality improvement processes into the Quality Assurance system. Specifically, through engagement in the State Quality Assurance Committee, it aimed to influence capacities for operationalisation of quality improvement methods recommended in the National Quality Assurance framework, and greater prioritisation of quality improvement during regular Quality Assurance monitoring and accreditation visits conducted by District Quality Assurance Managers. ACCESS participated in the recruitment of District Quality Assurance Managers and supported their quality improvement training through enrolment in the IHI Open School. It conducted analysis using data from hospitals participating in the collaborative programme, to differentiate process and infrastructure gaps required to achieve accreditation, and used this analysis to advocate for greater focus on process changes through continuous quality improvement efforts over infrastructure. ACCESS also participated in joint monitoring visits with District Quality Assurance Managers, and undertook external assessments during the accreditation process.

**6. Theory of change**

The Safe Care Saving Lives programme assumed that the reliable delivery of 20 evidence-based practices for intrapartum, early newborn care and care of small and sick newborns to prevent key drivers of newborn mortality, (i.e. birth asphyxia, complications of prematurity and newborn sepsis) would contribute to a reduction in newborn mortality at hospital level (see figure 3). This was in line with analysis indicating that increased coverage and quality of a package of interventions along the continuum of pre-conception, maternal and newborn care by 2025 could avert 71% of newborn deaths, and that the maximum effect on neonatal deaths is through interventions during labour and birth, followed by care of small and ill newborn babies ([15](#_ENREF_15)).

***Figure 3: Safe Care Saving Lives Drivers Diagram***


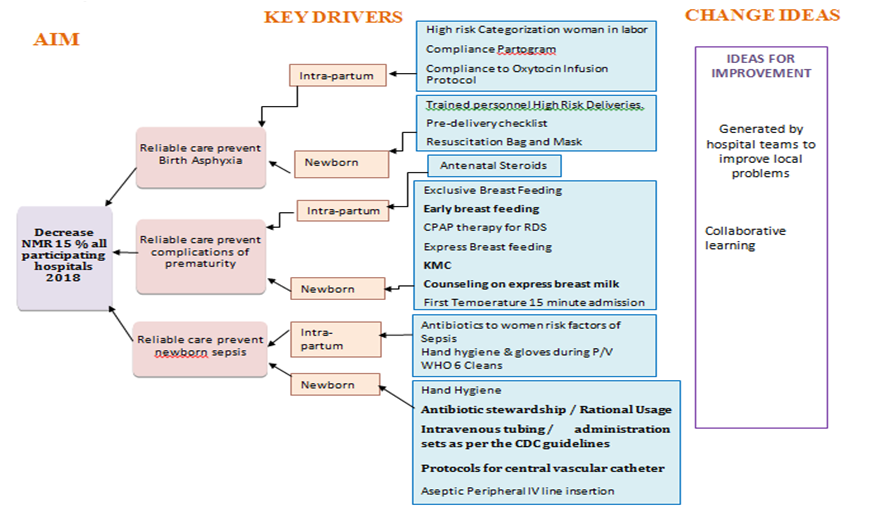


The programme aimed to improve the adoption of EBPs by supporting the establishment of a culture of quality improvement; the creation of new social norms promoting compliance with EBPs; and by improving cooperation across departments and cadres. It aimed to achieve capacity, behavioural and organisational level changes within each hospital, and it assumed that these would be enhanced by collaborative work, and supported by the local health system (Figure 4).

***Figure 4: Theory of change – Diagram 1: Levels of change***


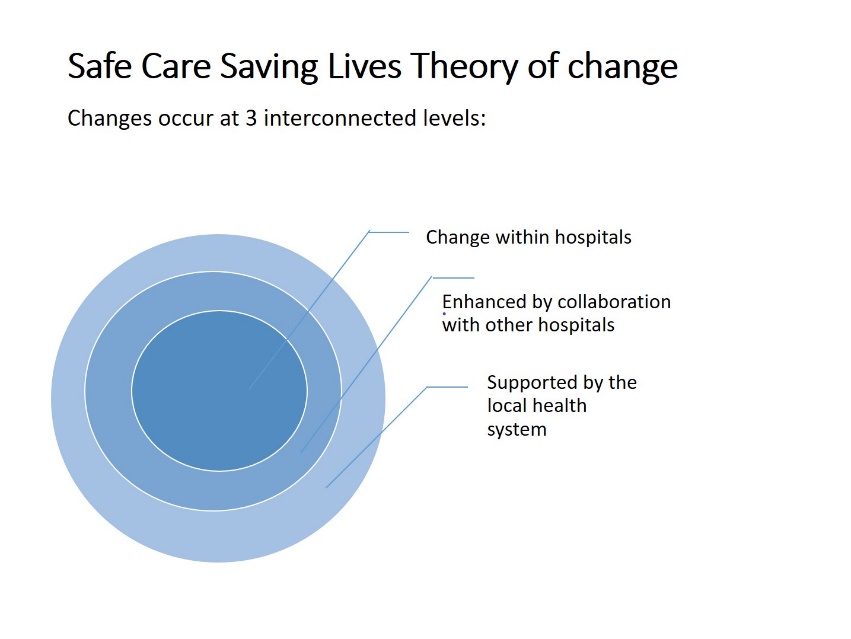


*5.1 Changes within each hospital*

At the core of the programme theory of change were active QI teams, regularly testing innovation, learning and improving, and supported by an engaged leadership focused on improvement. Therefore, the programme focused on formation of QI teams, capacity building, mentoring and support, to increase capability for continuous quality improvement, and to increase data literacy and capacity to use data in decisions, in both QI teams and hospital leaders. It also focused on sensitisation and continuous engagement of hospital leaders, to increase understanding on the need for quality improvement in relation to clinical outcomes and hospital strategic priorities, and to foster a perception that quality improvement is a feasible strategy, thus increasing their readiness to engage in quality improvement and commitment to the aim and methodology. These are illustrated in figure 5.

***Figure 5: Theory of change diagram 2 – Changes within hospitals***


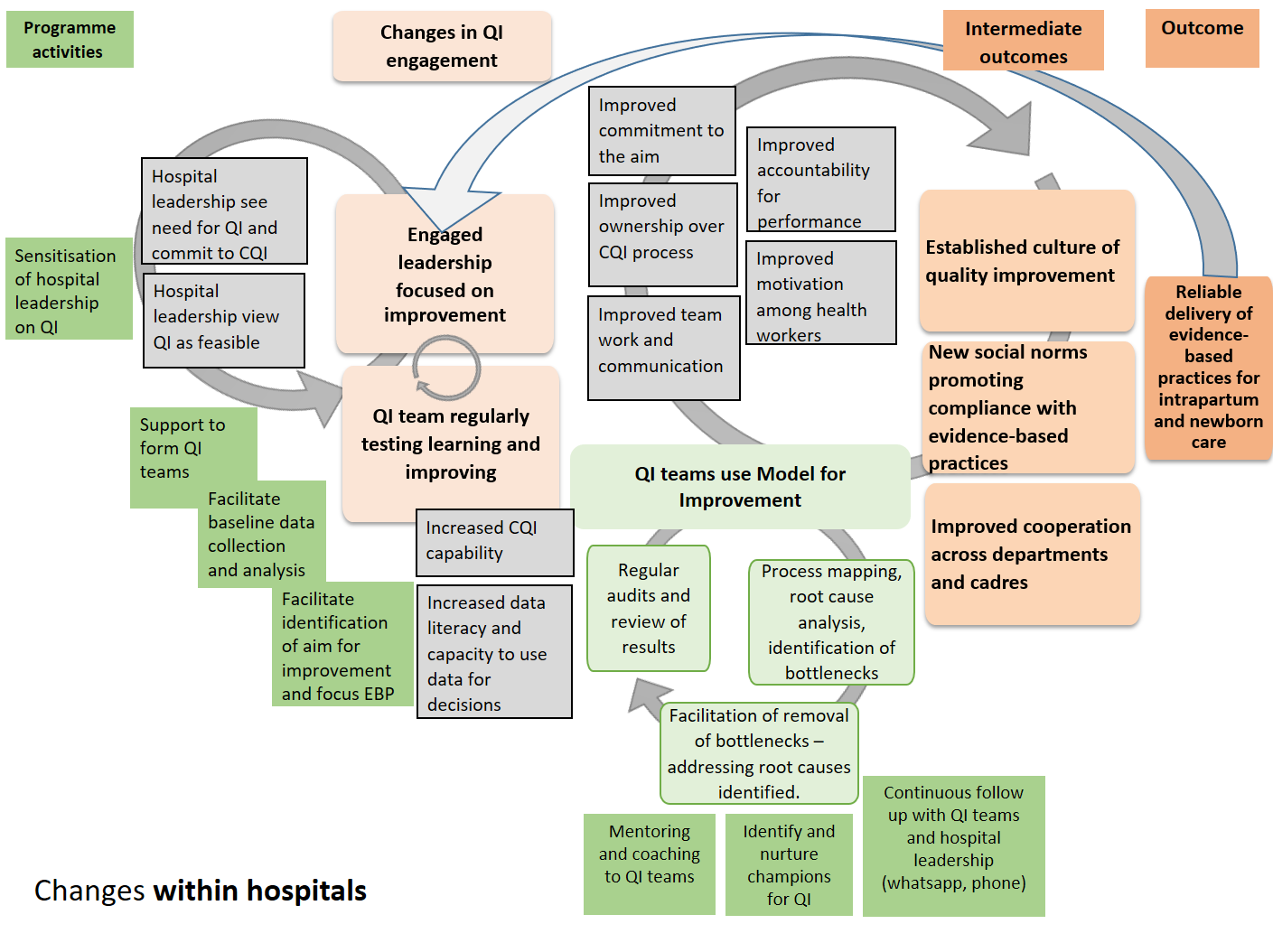


In line with evidence from published literature on quality improvement, engagement of leaders and active QI teams were thought of as a mutually reinforcing engine for change: the more QI teams demonstrated results through their work, the more leaders would increase their interest and engagement in quality improvement. An open leadership, focused on results and problem-solving, and providing the necessary motivation and resources for quality improvement was essential for effective quality improvement efforts ([7](#_ENREF_7), [16-18](#_ENREF_16)).

The combination of active QI teams, supported by engaged leaders, was assumed to be able to activate a variety of mechanisms leading to the establishment of a culture of improvement, the creation of new social norms and improved collaboration across teams and departments([19-21](#_ENREF_19)). The programme did not make explicit reference to theory, but its design echoed the behaviour change model at individual level, and social learning theory and normalisation process theory at organisational level ([22-24](#_ENREF_22)).

It should be noted that the identified mechanisms were also assumed to be mutually reinforcing, for example improved team work and communication would improve motivation among health workers, and vice versa. The programme assumed a plurality of pathways, given that each hospital could be considered a micro-cosmos of its own. Hence, the diagram in figure 5 represents potential conceptual avenues and not a sequential series of expected outcomes.

*5.2 Changes at Quality Improvement collaborative level*

The programme also assumed that changes within hospitals be enhanced by collaboration with other hospitals. The expectation was that the QI collaborative activities would generate rapid improvements in the clinical area of focus, by shortening the time for diagnosing problems and developing changes, and by providing an external stimulus to make large improvements by spreading of ideas across sites ([1](#_ENREF_1), [25](#_ENREF_25), [26](#_ENREF_26)).

The approach borrowed from Rogers’ diffusion of innovation theory, assuming that adoption of an innovation (a change idea for improvement) would be greater depending on *attributes of the idea itself*: the relative advantage over the status quo or other ideas that have been proposed; the *degree of compatibility with existing values*, experiences and needs; the *complexity of the idea*; its *trialability*, or the extent to which the idea had been tested; and its *observability*, i.e. the opportunity for people to observe the success of the change for others([27](#_ENREF_27)). This framework explained the focus on early adopters to showcase innovation and act as hubs for learning and improvement in mini-collaboratives.

The QI collaborative approach intended to enhance quality improvement efforts in various ways (see figure 6):

- For early adopter hospitals, it may provide reputational gains through recognition among peers, and further enhances team motivation and commitment to quality improvement ([19](#_ENREF_19)).
- For other hospitals participating in the QI collaborative, the collaborative approach may enhance leadership and frontline health workers’ engagement in quality improvement, by increasing perceptions of its feasibility (thus activating the observability mechanism, demonstrating the compatibility of the change with existing needs) and by favouring the diffusion of local innovation (thus activating the *trialability* mechanism)([12](#_ENREF_12), [28](#_ENREF_28)).

It also may reinforce the establishment of new social norms promoting compliance with EBPs, specifically by supporting the development of a culture of improvement across hospitals, which was assumed to generate normative pressures on other hospitals to adopt the quality improvement process and the focus practices, as in the long run they would find it damaging not to ([19](#_ENREF_19), [29](#_ENREF_29), [30](#_ENREF_30)).

***Figure 6: Theory of change diagram 3 – Changes at QI collaborative level***

**
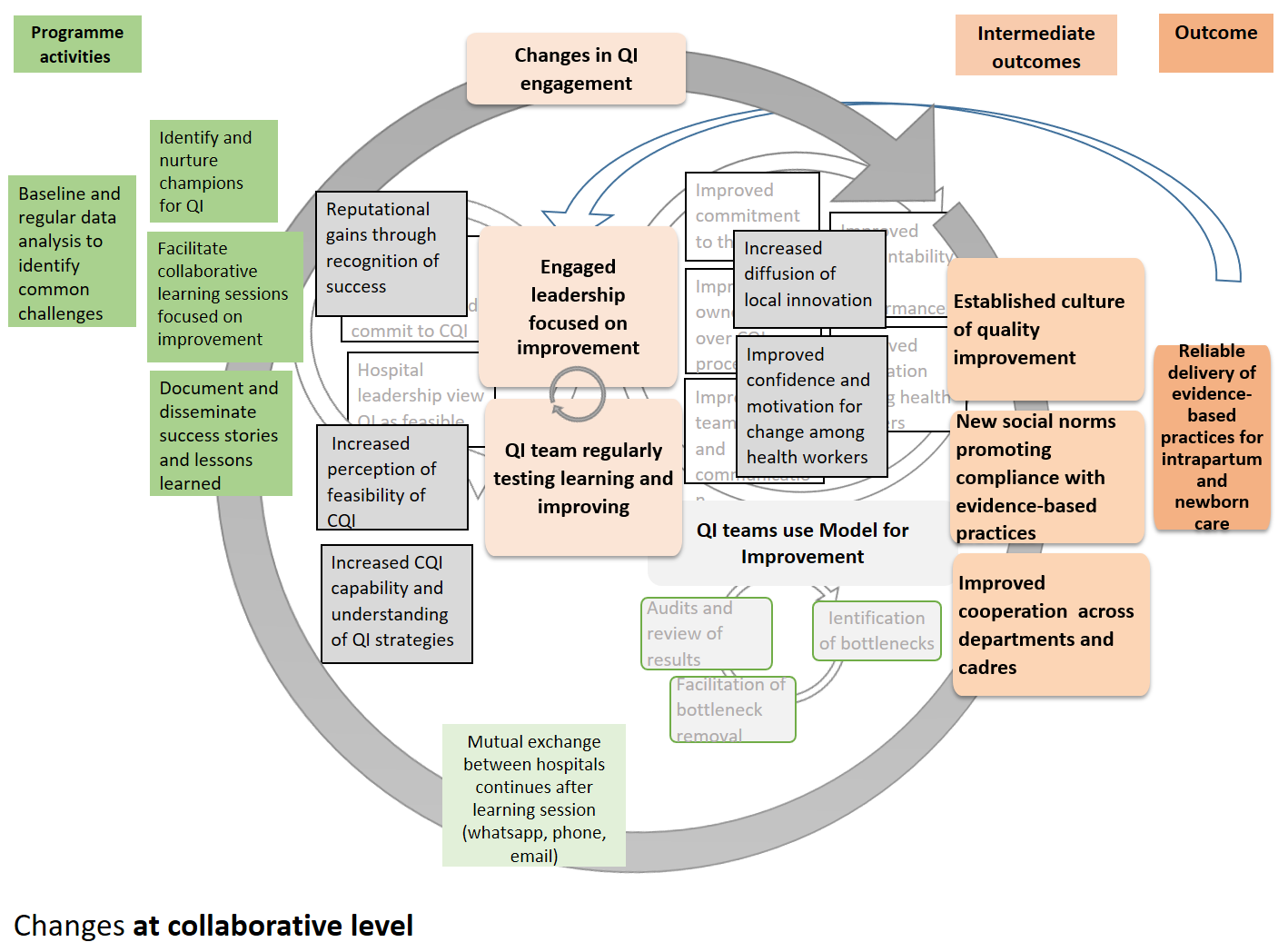
**

*5.3 Changes at health system level*

The programme embodied health systems theory in its core assumption that “every system is perfectly designed to achieve the results it obtains”([1](#_ENREF_1)), p. 79. The programme scale up strategy rested on the assumption that if a shift in prioritisation of quality improvement occurred among stakeholders that had a regulatory and governance function in the health system, this would trickle down to individual hospital leaders, and act as a facilitator of greater adoption of quality improvement processes and improved newborn care practices.

Although not explicitly articulated in its design, the programme’s positioning in the health system, and its activities with government-health insurance companies and with state and district level authorities emerged organically from programme experience in wave I, and in response to challenges in engaging hospital leadership. The intervention at health systems level focused on activating pressure on hospital leaders through mechanisms that the health systems literature describes as carrot, stick and sermon approaches, referring respectively to a system of financial or other types of incentives, coercive pressure, and normative or motivational pressures ([31](#_ENREF_31)) (see figure 7).

***Figure 7: Theory of change diagram 4 – Changes at the level of the health system***

**
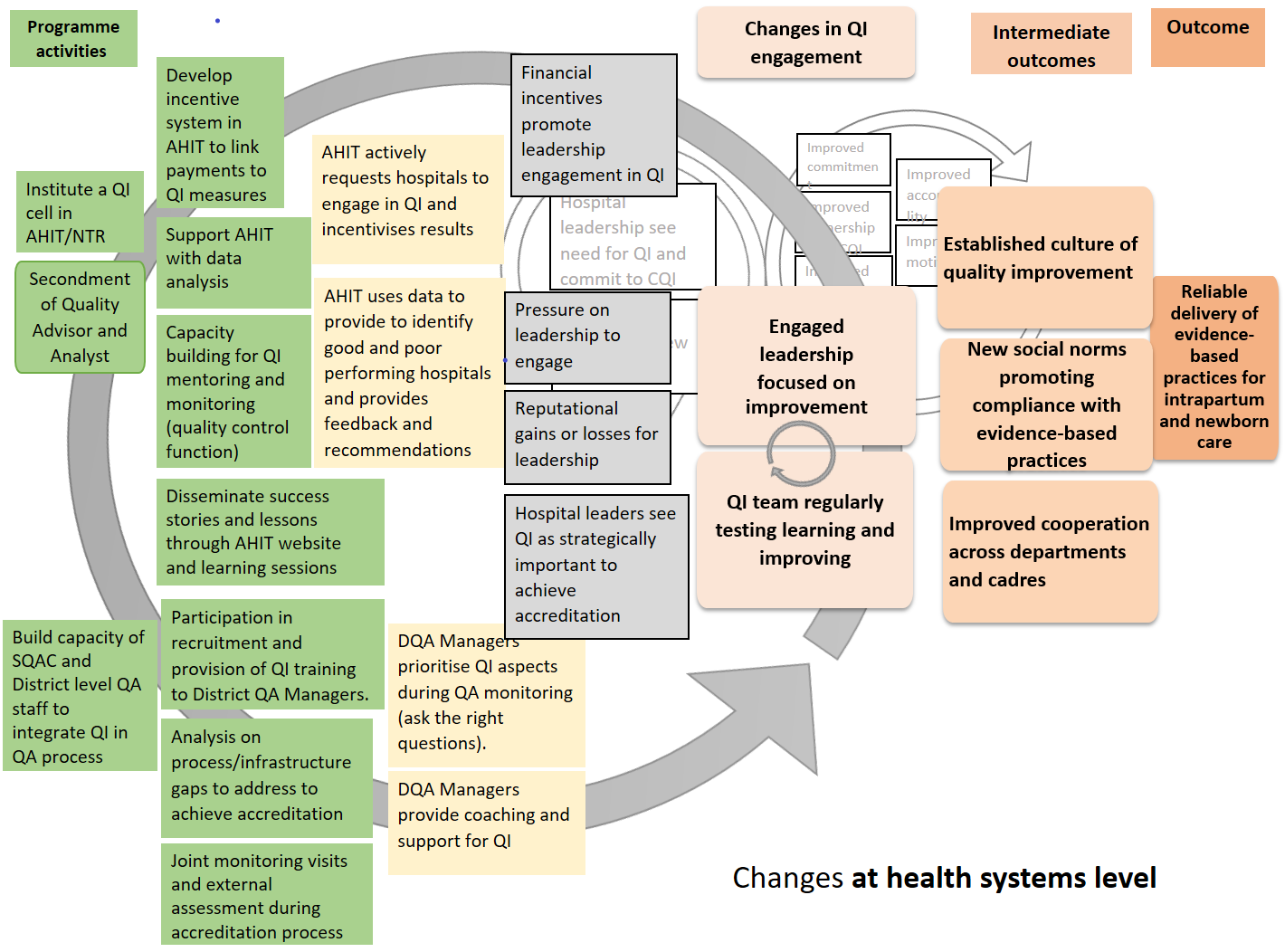
**

In the Safe Care Saving Lives programme, efforts to strengthen the incentive system included support to the development of a financial incentive system for quality improvement, linked to health insurance payments. When the programme started, Aarogyasri Health Care Trust gave incentives to hospitals that were accredited with the National Accreditation Board of Hospitals. ACCESS planned to work with the Aarogyasri Health Care Trust to expand the incentive system to link payments to hospitals adopting and continuing quality improvement, in addition to achieving accreditation, assuming that this would increase hospital leaders’ commitment and engagement in quality improvement.

Incentive-related pressure (carrot mechanisms) related to ACCESS advocating at state and district level for increased prioritisation of quality improvement in the quality assurance system. The programme assumed that if relevant Quality Assurance authorities in charge of monitoring, verification and accreditation “asked the right questions of leaders”, (that is, if they focused on process of care as opposed to inputs in their ongoing monitoring and assessments), hospital leaders would increasingly prioritise improvements in clinical practice within available resources, as these would be seen as strategically important towards accreditation. Accreditation would in turn increase clinicians’ professional recognition, hospital status, and potentially revenues.

Coercive pressures (also referred to as stick mechanisms) related to regulation, statutory powers, licencing, and in the case of health insurance companies, empanelment criteria. The assumed pathway by which Safe Care Saving Lives intended to activate coercive pressure was by strengthening health insurance companies’ capacity to directly request engagement in quality improvement activities by those empanelled, or to provide direct feedback or recommendation to hospitals. Essentially, the programme assumed that the health insurance companies would exert a quality control function in the system, which would activate coercive pressures on empanelled hospitals to conform to Aarogyasri Health Care Trust’s Standard Treatment Guidelines. This pathway rested on the ongoing analysis of hospital data to identify good and poor performing hospitals, on the regular discussion at the level of QI Cell on these data, and fundamentally, on the health insurance companies seeing quality improvement as a strategy for efficiency as well as effectiveness.

Normative and motivational pressures (also referred to as sermon mechanisms) related to promoting leadership engagement by nurturing personal commitment to the reduction of newborn mortality, the adoption of EBPs and a culture of quality improvement. The programme aimed to foster a networked community of leaders, committed to the programme aim and relentlessly focused on improvement. Relevant strategies included providing hospitals and clinicians with opportunities for reputational gains by sharing success stories, and cultivating a network of champions of quality improvement at all levels, through collaborative work.

**References**

1. Langley GJ, Moen R, Nolan KM, Nolan TW, Norman CL, Provost LP. The Improvement Guide: A Practical Approach to Enhancing Organizational Performance: John Wiley & Sons; 2009.

2. Twum-Danso NA, Dasoberi IN, Amenga-Etego IA, Adondiwo A, Kanyoke E, Boadu RO, et al. Using quality improvement methods to test and scale up a new national policy on early post-natal care in Ghana. Health Policy Plan. 2014;29(5):622-32.

3. Telangana Go. Aarogyasri Health Care Trust [25 October 2017]. Available from: <http://www.aarogyasri.telangana.gov.in/web/guest/aarogyasri-scheme>.

4. Access Health International. Safe Care Saving Lives Perinatal and Neonatal Collaborative Update Unpublished2014.

5. Twum-Danso NAY, Akanlu GB, Osafo E, Sodzi-Tettey S, Boadu RO, Atinbire S, et al. A nationwide quality improvement project to accelerate Ghanas progress toward Millennium Development Goal Four: design and implementation progress. International Journal for Quality in Health Care. 2012;24(6):601-11.

6. Kilo CM. A framework for collaborative improvement: lessons from the Institute for Healthcare Improvement's Breakthrough Series. Qual Manag Health Care. 1998;6(4):1-13.

7. Øvretveit J, Bate P, Cleary P, Cretin S, Gustafson D, McInnes K, et al. Quality collaboratives: lessons from research. Quality and Safety in Health Care. 2002;11(4):345-51.

8. Mittman BS. Creating the evidence base for quality improvement collaboratives. Annals of Internal Medicine. 2004;140(11):897-901.

9. Wilson T, Berwick DM, Cleary PD. What do collaborative improvement projects do? Experience from seven countries. The Joint Commission Journal on Quality and Patient Safety. 2003;29(2):85-93.

10. International AH. Safe Care Saving Lives Project. Quality Improvement Toolkit. 2016.

11. Berwick DM. The question of improvement. JAMA. 2012;307(19):2093-4.

12. de Silva D. Improvement collaboratives in health care. Evidence scan July 2014. London: The Health Foundation, 2014 No. 21.

13. Institute for Healthcare Improvement. Open School [6 December 2017]. Available from: <http://www.ihi.org/education/ihiopenschool/courses/Pages/default.aspx>.

14. National Health Mission MoHaFW, Government of India. National Quality Assurance Standards for public health facilities. New Delhi, India: Ministry of Health and Family Welfare, Government of India; 2016.

15. Bhutta ZA, Das JK, Bahl R, Lawn JE, Salam RA, Paul VK, et al. Can available interventions end preventable deaths in mothers, newborn babies, and stillbirths, and at what cost? Lancet. 2014;384(9940):347-70.

16. Duckers MLA, Wagner C, Vos L, Groenewegen PP. Understanding organisational development, sustainability, and diffusion of innovations within hospitals participating in a multilevel quality collaborative. Implementation Science. 2011;6.

17. Duckers MLA, Stegeman I, Spreeuwenberg P, Wagner C, Sanders K, Groenewegen PP. Consensus on the leadership of hospital CEOs and its impact on the participation of physicians in improvement projects. Health Policy. 2009;91(3):306-13.

18. Hulscher MEJL, Schouten LMT, Grol RPTM, Buchan H. Determinants of success of quality improvement collaboratives: what does the literature show? Bmj Quality & Safety. 2013;22(1):19-31.

19. Dixon-Woods M, Bosk CL, Aveling EL, Goeschel CA, Pronovost PJ. Explaining Michigan: Developing an Ex Post Theory of a Quality Improvement Program. Milbank Quarterly. 2011;89(2):167-205.

20. Pronovost PJ, Berenholtz SM, Goeschel C, Thom I, Watson SR, Holzmueller CG, et al. Improving patient safety in intensive care units in Michigan. Journal of Critical Care. 2008;23(2):207-21.

21. Horbar JD, Plsek PE, Leahy K, Nic/Q. NIC/Q 2000: establishing habits for improvement in neonatal intensive care units. Pediatrics. 2003;111(4 Pt 2):e397-410.

22. Grol R, Grimshaw J. From best evidence to best practice: effective implementation of change in patients' care. Lancet. 2003;362(9391):1225-30.

23. Rowe AK, de Savigny D, Lanata CF, Victora CG. How can we achieve and maintain high-quality performance of health workers in low-resource settings? The Lancet. 2005;366(9490):1026-35.

24. May C, Finch T. Implementing, embedding, and integrating practices: an outline of normalisation process theory. Sociology. 2009;43.

25. Barker PM, Reid A, Schall MW. A framework for scaling up health interventions: lessons from large-scale improvement initiatives in Africa. Implementation Science. 2016;11.

26. Singh K, Brodish P, Speizer I, Barker P, Amenga-Etego I, Dasoberi I, et al. Can a quality improvement project impact maternal and child health outcomes at scale in northern Ghana? Health Research Policy and Systems. 2016;14(1):45.

27. Rogers EM. Diffusion of Innovation. 5th edition ed. London: Simon & Schuster; 2013.

28. Duckers MLA, Groenewegen PP, Wagner C. Quality improvement collaboratives and the wisdom of crowds: spread explained by perceived success at group level. Implementation Science. 2014;9.

29. Carter P, Ozieranski P, McNicol S, Power M, Dixon-Woods M. How collaborative are quality improvement collaboratives: a qualitative study in stroke care. Implementation Science. 2014;9(1):32.

30. Dainty KN, Scales DC, Sinuff T, Zwarenstein M. Competition in collaborative clothing: a qualitative case study of influences on collaborative quality improvement in the ICU. BMJ Quality & Safety. 2013;22(4):317-23.

31. Bennett S, Dakpallah G, Garner P, Gilson L, Nittayaramphong S, Zurita B, et al. Carrot and Stick - State Mechanisms to Influence Private Provider Behavior. Health Policy and Planning. 1994;9(1):1-13.
