# Supplementary material for: Evaluation of the Safe Care, Saving Lives (SCSL) quality improvement collaborative for neonatal health in Telangana and Andhra Pradesh, India: a study protocol
Source: Glob Health Action. 2019 Mar 8;12(1):1581466. doi: 10.1080/16549716.2019.1581466 (PMC6419630; doi:10.1080/16549716.2019.1581466)
Supplement: Supplemental Material [file ZGHA_A_1581466_SM0324.zip › Annex file C.docx]

Web annex C: List of indicators for quantitative evaluation

| **Indicator** | **Tool** | **Evidence-based practices** | **Comment** |
| --- | --- | --- | --- |
| **Impact indicators** |  |  |  |
| % of stillbirth of all hospital deliveries | Labour room register | Reliable intra-partum care and newborn resuscitation |  |
| % of neonates dying before the age of 7-days / 28-days among those admitted to the newborn care unit | Neonatal care register  Telephonic Interviews with mothers after discharge | Prevent complications from prematurity and neonatal sepsis prevention |  |
| **Output indicators** |  |  |  |
| % of high-risk admissions correctly flagged | Case note abstraction | High risk categorization |  |
| % of admissions where essential information was documented in partograph and attached to case notes | Case note abstraction | Compliance with partogram |  |
| % of all induced deliveries where use of oxytocin protocol was indicated on case notes | Case note abstraction | Compliance to oxytocin infusion protocol | Sample size will be too small to measure effect |
| % of high risk deliveries where personnel trained in resuscitation were present | Observation of delivery | Trained personnel at high risk delivery | Sample size will be too small to measure effect |
| % of admissions where safe childbirth checklist used and attached to case notes | Case note abstraction | Pre-delivery checklist |  |
| % of asphyxiated babies for which resuscitation was initiated within 1 minute | Observations of delivery | Resuscitation with bag and mask | Sample size will be too small to measure effect |
| No indicator |  | Antenatal steroids | Sample size will be too small for meaningful indicator |
| % of babies discharged from newborn care unit who were exclusively breastfed at first interview after discharge | Telephonic Interviews with mothers after discharge | Exclusive breastfeeding |  |
| % of babies seen in the neonatal care admission ward for whom temperature was measured within 15 minutes | Observations of admissions | First temperature measurement as admission to neonatal care unit |  |
| No indicator |  | Delivery room CPAP | Sample size will be too small for meaningful indicator |
| % of mothers with risk of sepsis where antibiotics were given | Case note abstraction | Antibiotics to women with risk factors of sepsis | Not assessed at baseline |
| % of vaginal examinations where hygiene standards are met | Observations | Hand hygiene & gloves during vaginal examinations |  |
| % of deliveries where the six cleans were adhered to | Observations | WHO six cleans |  |
| % of patient contacts where hygiene standards are met | Observations of patient contact in newborn care unit | Hand hygiene |  |
| % of cannulations and i.v. line insertion where hygiene standards are met | Observations of iv line | Aseptic peripheral cannulation and iv line insertion |  |
| % babies admitted to a newborn care unit for prematurity for whom the mother reports Kangaroo Mother Care” |  | Kangaroo Mother care |  |
| No indicator |  | Aseptic central i.v- line insertion | Rare event  Sample size will be too small to measure effect |
| **Others** |  |  |  |
| Days of stay in newborn care unit | Neonatal care register | Neonatal sepsis prevention |  |
| % of inborn babies referred to neonatal care unit because of asphyxia | Neonatal care register | Reliable intra-partum care and newborn resuscitation |  |
